# Cerebrospinal fluid cells immune landscape in multiple sclerosis

**DOI:** 10.1186/s12967-021-02804-7

**Published:** 2021-03-25

**Authors:** Zijian Li, Yongchao Liu, Aili Jia, Yueran Cui, Juan Feng

**Affiliations:** Department of Neurology, Shengjing Hospital of China Medical University, No. 36 Sanhao Street, Heping District, Shenyang, Liaoning, 110004 China

**Keywords:** Multiple sclerosis, Bioinformatics analysis, CIBERSORT, Immune microenvironment

## Abstract

**Background:**

Multiple Sclerosis (MS) is a potentially devastating autoimmune neurological disorder, which characteristically induces demyelination of white matter in the brain and spinal cord.

**Methods:**

In this study, three characteristics of the central nervous system (CNS) immune microenvironment occurring during MS onset were explored; immune cell proportion alteration, differential gene expression profile, and related pathways. The raw data of two independent datasets were obtained from the ArrayExpress database; E-MTAB-69, which was used as a derivation cohort, and E-MTAB-2374 which was used as a validation cohort. Differentially expressed genes (DEGs) were identified by the false discovery rate (FDR) value of < 0.05 and |log2 (Fold Change)|> 1, for further analysis. Then, functional enrichment analyses were performed to explore the pathways associated with MS onset. The gene expression profiles were analyzed using CIBERSORT to identify the immune type alterations involved in MS disease.

**Results:**

After verification, the proportion of five types of immune cells (plasma cells, monocytes, macrophage M2, neutrophils and eosinophils) in cerebrospinal fluid (CSF) were revealed to be significantly altered in MS cases compared to the control group. Thus, the complement and coagulation cascades and the systemic lupus erythematosus (SLE) pathways may play critical roles in MS. We identified NLRP3, LILRB2, C1QB, CD86, C1QA, CSF1R, IL1B and TLR2 as eight core genes correlated with MS.

**Conclusions:**

Our study identified the change in the CNS immune microenvironment of MS cases by analysis of the in silico data using CIBERSORT. Our data may assist in providing directions for further research as to the molecular mechanisms of MS and provide future potential therapeutic targets in treatment.

**Supplementary Information:**

The online version contains supplementary material available at 10.1186/s12967-021-02804-7.

## Background

MS is one of the most common inflammatory demyelinating neurological diseases, and the disability resulting from it presents a huge social burden, putting many young adults in wheelchairs from an early age, and causing a range of problems for the family of the affected person. Though it is not actually a malignancy, MS is also sometimes known as one of the ‘nonfatal cancers’. To date, there is no medical therapeutic strategy that can cure MS. In the initial stages of MS, and also in an experimental autoimmune encephalomyelitis (EAE) animal model, following damage to the blood–brain barrier (BBB), inflammatory cells infiltrate tissues of the brain and spinal cord. Persistent inflammatory cells and cytokines in the CNS microenvironment caused by this loss of BBB integrity can promote disease progression and recurrence. Indeed, the extent of BBB permeability, which disrupts the homeostasis of the CNS immune microenvironment, is directly correlated with disease severity [1]. According to recent reports in literature, the cells of the immune system such as M1 macrophage/microglia [2, 3], Th1 cell [4, 5], Th17 cell [6] play vital roles in exacerbating the MS disease, however, M2 macrophage/microglia [7], Th2 cell [8], regulatory T cell [9] play vital roles in ameliorating the MS disease. Furthermore, many different types of immunocytes such as B cell [10, 11], neutrophil [12], dendritic cell [13] and mast cell [14] were involved in the pathogenesis, development or relapse of MS.

CSF is a plasma-like liquid that circulates in the ventricles and sub-arachnoid space, providing the brain with nutrient delivery, waste removal, and protection from mechanical injury. Because of the ventricular neuroanatomy and the characteristics of the circulation and production of CSF, there is frequently a corresponding relationship between the CSF laboratory findings and the pathological changes of CNS. Thus CSF cytology and biochemistry is an important basis for the diagnosis of disease of brain tissue. For example, CSF laboratory tests may reveal the existence and nature of radiculopathy in the subarachnoid space, meningeal disease and inflammatory lesions of brain parenchyma. A ‘liquid biopsy’ of CSF may also be useful for detecting nervous system tumors. The detection of oligoclonal bands (OCBs), anti-myelin basic protein (MBP) antibody, and anti-myelin oligodendrocyte glycoprotein (MOG) antibody in the CSF and serum are now important diagnostic markers in the diagnosis of MS and are widely used clinically in diagnosis. However, more research is needed to fully understand how these CSF markers change over the disease progression. Therefore, it is essential and important to undertake a comprehensive analysis of the CNS immune microenvironment, of differentially expressed genes, signaling pathways, and changes in the composition of immune cells in MS, compared to those of a normal control immune microenvironment. Such analysis may provide exciting new insights in understanding normal CSF homeostasis and the pathological changes in MS.

Whether MCPcounter, TIminer or other scoring methods based on labeled genes are used, or CIBERSORT, TIMER, ImmuCellAI and other scoring methods based on cell mixture deconvolution expression characteristics, it is gene expression data that quantify the immune cell proportion. CIBERSORT [15] can quantitatively calculate the abundance of specific cell types in complex tissues, and its results have been verified by fluorescence activated cell sorting. In recent years, research into the analysis of immune microenvironment cell types has progressed, with researchers developing new methods such as CIBERSORTx [16] and xCell [17]. The analysis of immune cell subtype distribution patterns has proved of great value and has been used in many kinds of tumors [18, 19], and immune related diseases [20, 21]. However, until now, no CIBERSORT analysis of the immune cell subtype distribution pattern associated with MS has been undertaken based on CSF samples. The disease lesions, immunocytes in CSF, and the CSF supernatant which bathing the CNS tissue constitute the immune microenvironment of the MS disease together. Previous studies mainly focused on the gene expression change in CSF supernatant or the immunocytes infiltrated alteration in brain tissue lesions. This study focused on CIBERSORT analysis based on the gene transcriptional matrix of the CSF cells of both MS cases and control groups in order to find a missing link to complete the whole picture of the immune microenvironment. Moreover, exploring the changes in cell composition and gene expression levels of cells in the CSF, a part of the CNS immune microenvironment, will help us better understand the detail of the processes occurring during disease.

In this study, we explored the proportion of the immune cell types in the CSF of individuals from two microarray datasets using the CIBERSORT method, and performed a comprehensive analysis of related immune cells, genes and signaling pathways. The raw data of datasets E-MTAB-2374 and E-MTAB-69 were obtained from the EBI ArrayExpress database (https://www.ebi.ac.uk/arrayexpress), which stores data from high-throughput functional genomics experiments and makes the data available to the research community. The study of drug pathways targeting disease-related immune cells and genes will assist the development of new diagnosis and treatment strategies for MS.

## Materials and methods

### Microarray datasets collection

The filter search inclusion criteria were as follows: (1) search term, multiple sclerosis; (2) organism, Homo sapiens; (3) type, transcription profiling by array; and (4) dataset including MS cases and control CSF samples. The exclusion criteria were as follows: (1) dataset containing fewer than 10 MS samples and 10 control samples; (2) the profile was based on cell lines; (3) individuals who received immunomodulatory drugs; and (4) individuals with other neurological diseases of a non-inflammatory kind were used as the control population. There was no dataset in the GEO database that met the inclusion criteria. In this study, the EBI ArrayExpress functional genomics database (https://www.ebi.ac.uk/arrayexpress/) was used to acquire the gene expression profiles of MS.

**Fig. 1:**
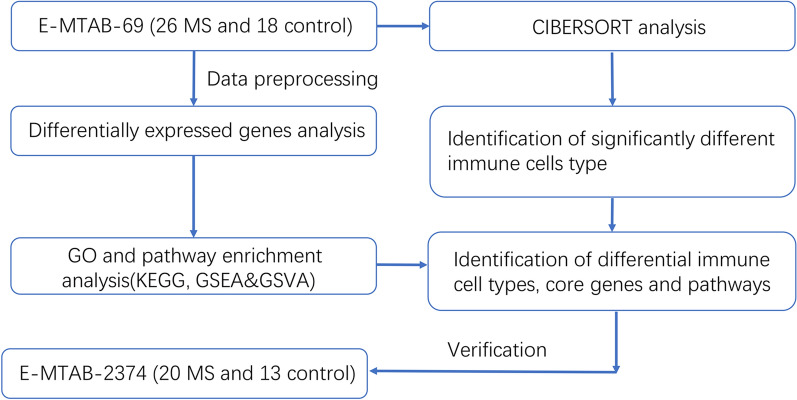
The flow chart of the analysis procedure. MS, multiple sclerosis; CIBERSORT, cell-type identification by estimating relative subsets of RNA transcripts; GO, Gene Ontology; KEGG, Kyoto Encyclopedia of Genes and Genomes; GSEA, gene set enrichment analysis; GSVA, gene set variation analysis

The raw data of E-MTAB-69 [22] was used to obtain the derivation dataset, which included 18 other non-inflammatory neurological disorders as controls and 26 MS CSF samples. The raw data of E-MTAB-2374 [23] was used as the validation dataset, which included 13 other neurological diseases as controls and 35 MS CSF samples (15 patients who received immunomodulatory drugs were excluded, so that only 20 MS samples were finally included for further analysis). The detailed information of MS cases and control samples in these two datasets were listed in the Additional file 4: Table S1. The samples in the two datasets were detected by the array of A-AFFY-44—Affymetrix GeneChip Human Genome U133 Plus 2.0 [HG-U133_Plus_2]. Figure 1 shows the details of the study process.

### Data preprocessing and DEG analysis

The raw expression profile data of these two datasets was downloaded from the EBI ArrayExpress database in CEL format (as.cel files). Bioinformatics analysis was performed using the R version 3.6.3 software. Data normalization and background correction were performed by the R package ‘affy’. Next, we converted the probe level data into gene expression values. If multiple probes corresponded to the same gene, we took the average expression value as the gene expression value. Differentially Expressed Genes (DEGs) between the MS and control groups were determined using the R package ‘limma’. Significance analysis of the microarray data was performed, with the selection criteria as follows: (1) false discovery rate (FDR) value < 0.05; (2) |log2 (Fold Change)|> 1 (fold change > 2 or < 0.5). Moreover, we used STRING and Cytoscape software version 3.8.0 to construct the PPI network.

### GO functional and KEGG pathway enrichment analysis

The ‘clusterProfiler’ package in R was used to determine the biological functions of DEGs, which identified Gene Ontology (GO) biological process (BP), cellular components (CC), molecular function (MF) and KEGG (Kyoto Encyclopedia of Genes and Genomes) pathway enrichment analyses. The cutoff criterion for the GO and KEGG pathway analysis were both set at adjusted p value < 0.05.

### Gene set variation analysis (GSVA) and gene set enrichment analysis (GSEA)

In this study, the open source ‘GSVA’ package for R was used to estimate variation of pathway activity over a sample population in an unsupervised manner [24], based on the microarray data. Furthermore, the ‘limma’ package for R was used to build linear models for comparing GSVA scores between MS cases and the control group. The cutoff criteria for GSVA were set as adjusted p value < 0.05 and |log2(fold change) |≥ 0.2. Furthermore, GSEA software was used to identify differentially enriched pathways between MS cases and the control groups with significant differences. The previously annotated gene set c2.cp.kegg.v7.1.symbols.gmt was chosen as the reference gene list. The cutoff value for the GSEA was set as p value < 0.05.

### Immune cell landscape analysis

CIBERSORT is a deconvolution algorithm that converts a normalized gene expression matrix into a constitutive distribution pattern of immune cells. In this study, we used the dataset E-MTAB-69 containing 26 MS cases and 18 controls to estimate the ratios of 22 types of infiltrated immune cells at the onset of disease. The immune cell types were listed as follows: neutrophils, monocytes, eosinophils, M0 macrophages, M1 macrophages, M2 macrophages, resting NK cells, activated NK cells, resting dendritic cells, activated dendritic cells, resting mast cells, activated mast cells, naïve CD4 + T cells, memory activated CD4 + T cells, memory resting CD4 + T cells, CD8 + T cells, regulatory T cells, γδ T cells, follicular helper T cells, naïve B cells, and memory B cells. For each sample, the summation of the total of each of the 22 types of immune cells' evaluated ratio was 100%. The CIBERSORT estimation result (with p value < 0.05) was thus taken as an accurate prediction of the immune cell composition. The samples that met the constraints were selected for further analysis.

### Validation of core genes, immune cells, and pathways

The microarray data of 13 controls and 20 MS samples (without immunomodulatory treatment) from the E-MTAB-2374 dataset was used to verify the findings in the E-MTAB-69 derivation dataset. We examined the expression of core genes, immune cell alterations and DEGs enrichment pathways. A p value < 0.05 was considered to indicate a statistically significant difference. The intersection of the findings between the two datasets was considered to be real core genes, cells, and pathways.

Furthermore, we used an online digital algorithm tool—Immune Cell Abundance Identifier (ImmuCellAI) (http://bioinfo.life.hust.edu.cn/ImmuCellAI#!/) [25], a gene set signature-based method calculated using the single sample gene set enrichment analysis (ssGSEA), to validate CIBERSORT computational analysis data. ImmuCellAI is a tool to estimate the abundance of 24 immune cells from gene expression dataset including RNA-Seq and microarray data, in which the 24 immune cells are comprised of 18 T-cell subtypes and 6 other important immune cells: B cell, NK cell, Monocyte, Macrophage, Neutrophil and DC.

### Statistical analysis

Statistical analysis and graphs were performed using R software, version 3.6.2 (the R Foundation for Statistical Computing). A p value < 0.05 was considered to be statistically significant.

## Results

### Identification of core DEGs

**Fig. 2:**
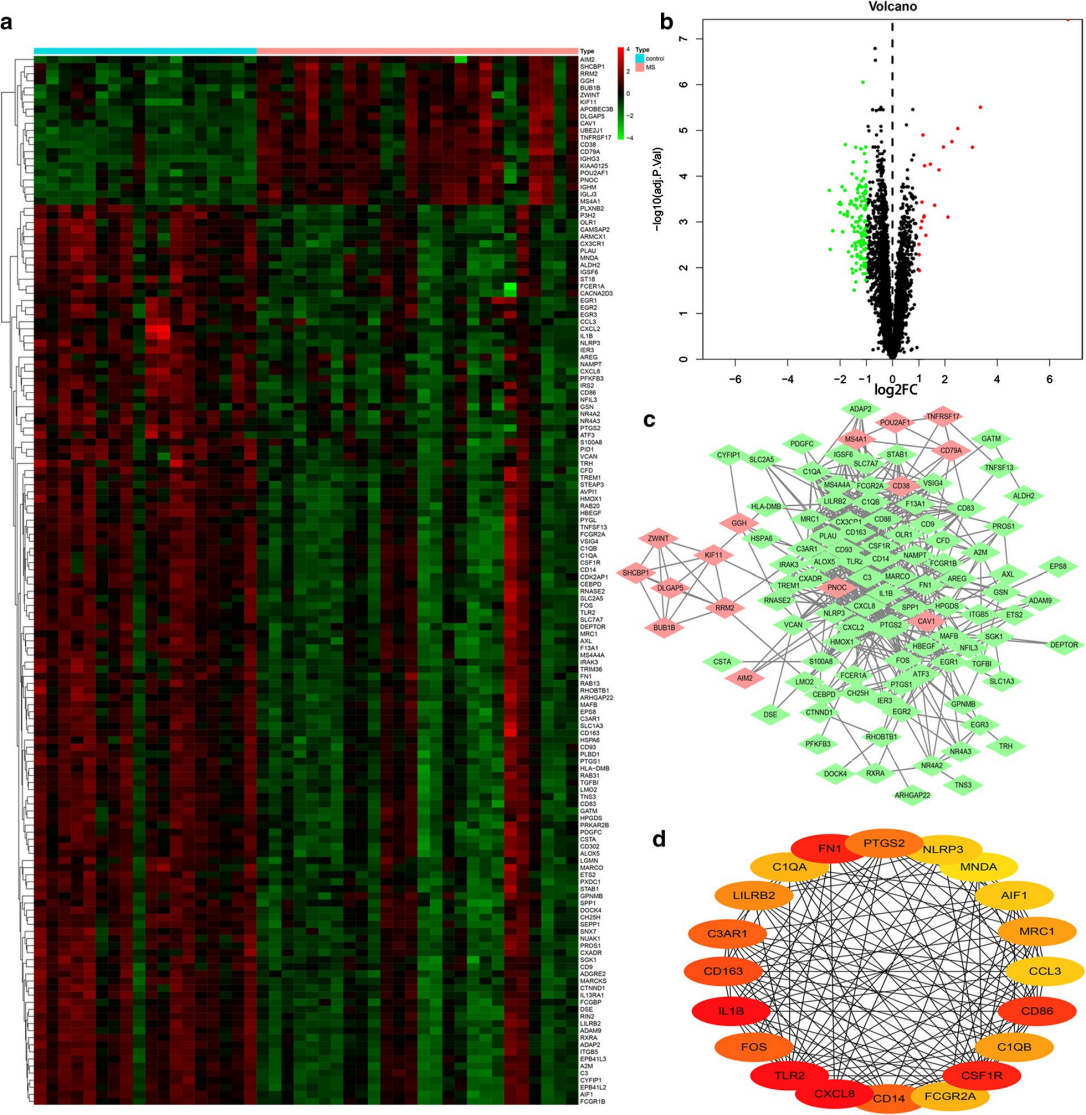
Identification and analysis of differentially expressed genes of dataset E-MTAB-69. **a** Heatmap of 148 DEGs between MS cases and control group. DEGs, differentially expressed genes. Green means downregulated; red means upregulated. **b** Volcano plot showed 148 DEGs between MS cases and control group. Green points represent relatively downregulated genes, red points represent upregulated genes, black points represent genes showing no significant alteration. **c** PPI network of DEGs between MS cases and control group. **d** The top 20 nodes ranked by Degree algorithm calculated by Cytohubba plugin in Cytoscape software

The hierarchical cluster analysis heatmap showed significantly different distributions of gene expression patterns between MS cases and control samples of dataset E-MTAB-69 (Fig. 2a). Under the threshold values of FDR < 0.05 and |log2 (Fold Change)|> 1, a total of 148 DEGs (21 up-regulated and 127 down-regulated) were chosen for further analysis, as shown in Fig. 2b and Additional file 4: Table S2. The interactions among the 148 DEGs were visualized in the PPI network. We identified 111 nodes and 592 edges among the DEGs and used the Cytoscape software platform for visualization (Fig. 2c). Genes with the top 20 degree scores based on cytoHubba analysis were identified as core genes (Table 1 and Fig. 2d). The degree means a connectivity degree that a gene connects with other genes in the PPI network. For example, degree = 1 means the connection comes from this gene or ends up with this gene. The closer the relationship with other genes in the network, the higher the degree value of the gene.

**Table 1:** The expression analysis of the top 20 hub genes with the highest interaction degree

Gene symbol	LogFC	P.Value	Adj. P.Value	Degree	Up/down-regulated
TLR2	− 1.402720222	3.46E−05	0.000996373	38	Down-regulated
CXCL8	− 1.991659081	1.66E−05	0.000623919	38	Down-regulated
IL1B	− 1.353392774	5.32E−06	0.000322284	38	Down-regulated
FN1	− 1.534988876	2.41E−05	0.000796163	34	Down-regulated
CSF1R	− 1.212137423	5.36E−05	0.001354478	34	Down-regulated
CD86	− 1.086235688	0.000162393	0.002835159	32	Down-regulated
CD163	− 1.345148553	0.000387759	0.005193712	29	Down-regulated
CD14	− 1.899109363	7.01E−05	0.001644275	28	Down-regulated
C3AR1	− 1.333582312	6.91E−05	0.001631076	28	Down-regulated
FOS	− 1.088599141	0.000691142	0.007925829	28	Down-regulated
PTGS2	− 1.585185487	5.97E−06	0.000348936	26	Down-regulated
LILRB2	− 1.260703038	2.49E−05	0.000803783	25	Down-regulated
C1QB	− 1.677793359	0.000312054	0.00446939	23	Down-regulated
MRC1	− 1.399146821	0.000212776	0.00339139	23	Down-regulated
C1QA	− 1.395713692	0.002766721	0.020479401	22	Down-regulated
FCGR2A	− 1.580725252	7.77E−06	0.000400066	22	Down-regulated
AIF1	− 1.043775506	5.30E−07	7.89E−05	21	Down-regulated
NLRP3	− 1.127686613	3.53E−10	8.93E−07	21	Down-regulated
CCL3	− 1.113448799	0.000134268	0.00250386	21	Down-regulated
MNDA	− 1.032416671	3.19E−05	0.000952134	20	Down-regulated

*Down-regulated* down-regulated in MS cases

### GO and KEGG analysis

**Fig. 3:**
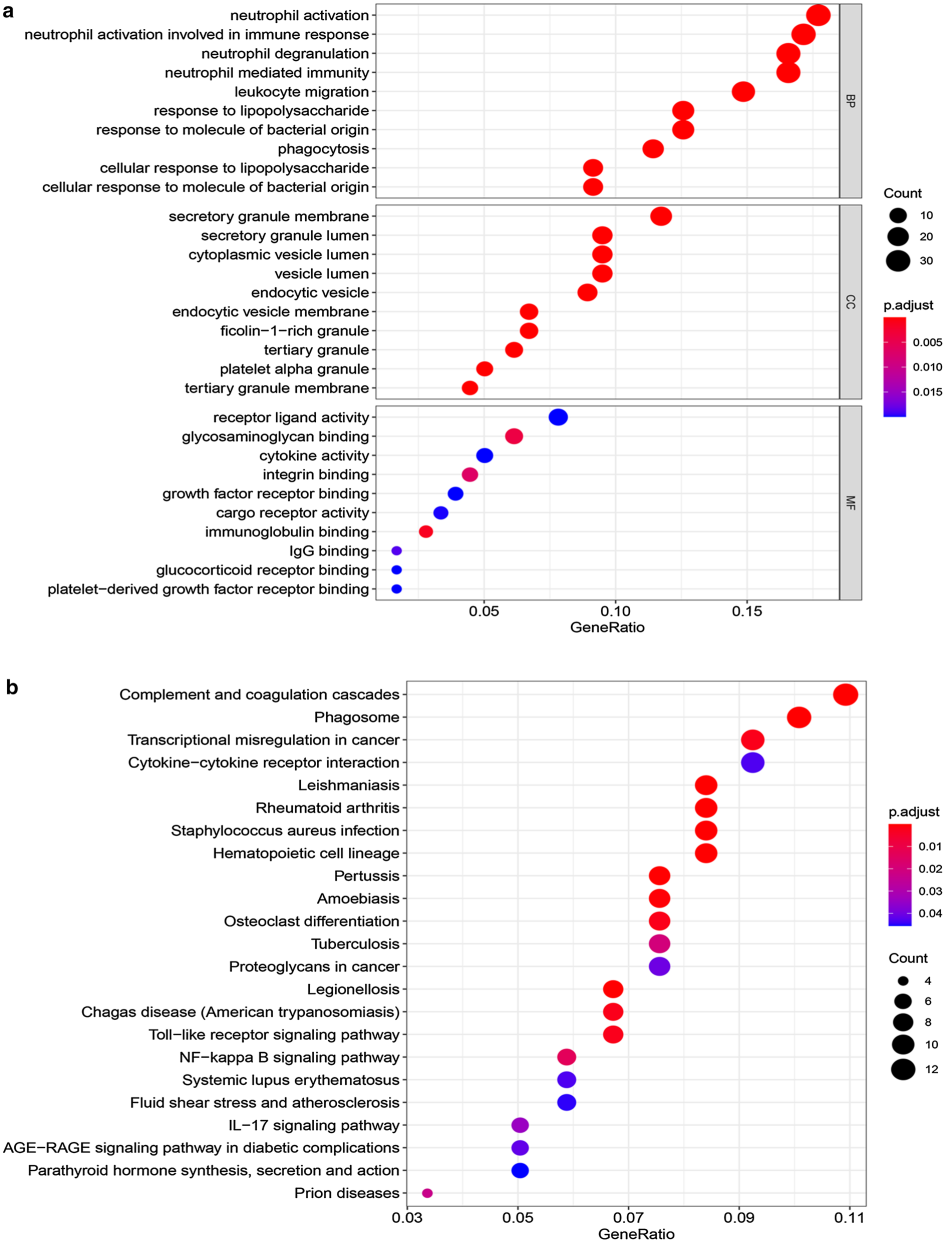
The GO and KEGG pathway analysis of dataset E-MTAB-69. **a** Bubble plot of GO gene set enrichment analysis of among all the DEGs (top 10 of BP, CC and MF). GO, Gene Ontology; BP, biological process; CC, cellular components; MF, molecular function. **b** Bubble plot of KEGG gene set enrichment analysis of among all the DEGs. Gene ratio: the ratio of the enriched genes to the total number of genes in the relative pathway in the database. KEGG, Kyoto Encyclopedia of Genes and Genomes. Count: the DEGs number enriched in each pathway

Then, we performed the GO and KEGG analyses to further explore the pathways in which DEGs were enriched of dataset E-MTAB-69. The GO analysis results showed that DEGs were mainly enriched in neutrophil activation, neutrophil activation involved in immune response, neutrophil degranulation, neutrophil mediated immunity and leukocyte migration, etc. The detailed top ten GO (BP, CC and MF) annotation terms are shown in Fig. 3a. The KEGG pathways of the DEGs are shown in Fig. 3b, which were mainly enriched in pathways of complement and coagulation cascades, phagosomes, transcriptional misregulation in cancer, cytokine-cytokine receptor interaction, Leishmaniasis and so on. Most of these pathways were associated with immune and inflammatory responses.

### GSVA and GAEA analysis

**Fig. 4:**
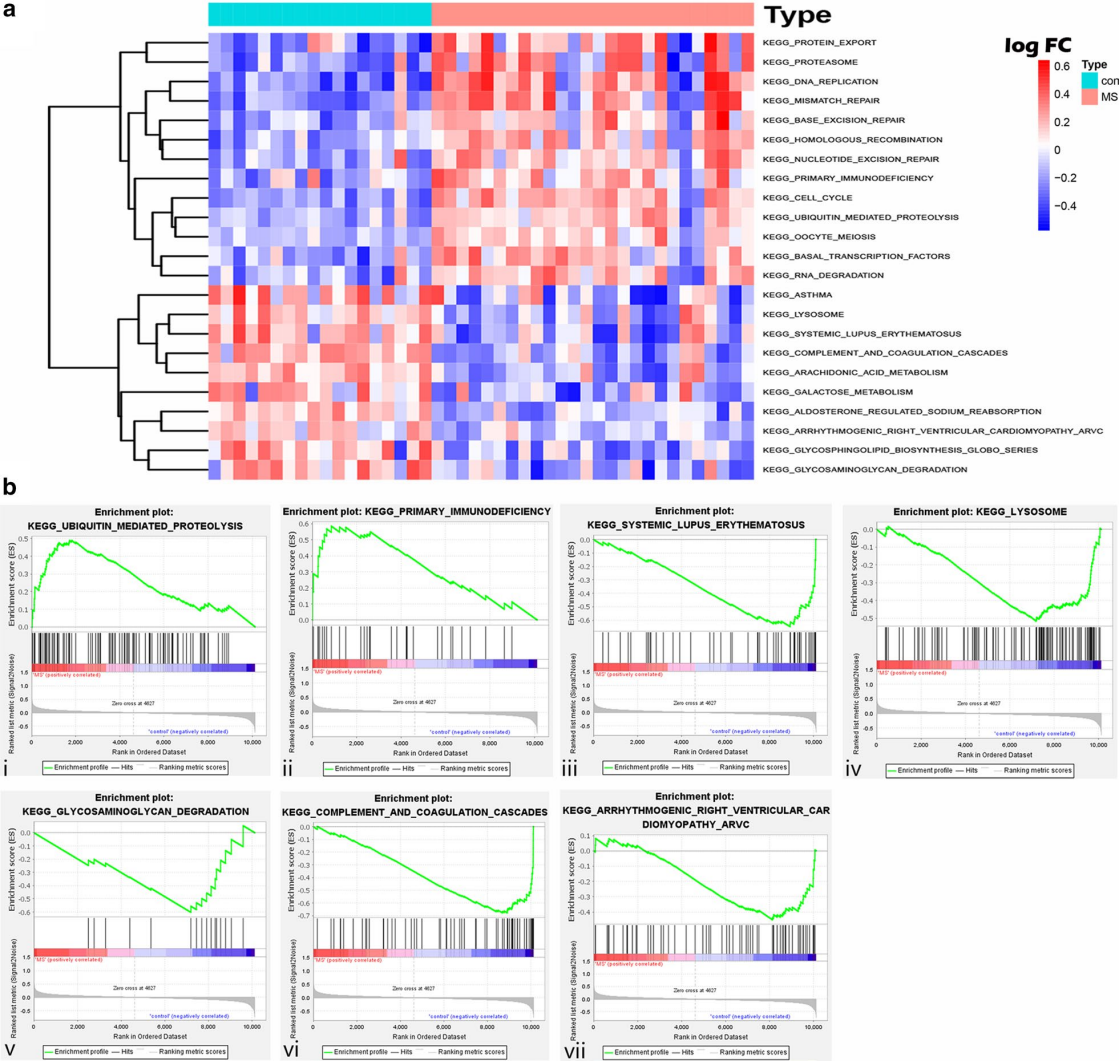
GSVA and GSEA of MS cases and controls of dataset E-MTAB-69. **a** Heatmap of GSVA scores of the KEGG gene-set enriched in samples of derivation dataset. **b** GSEA results of pathways which overlapped with results of GSVA in the derivation dataset. GSEA, gene set enrichment analysis; GSVA, gene set variation analysis; MS, multiple sclerosis; con, control group

GSVA results of dataset E-MTAB-69 showed that 13 pathways were significantly activated in MS, whereas 10 were inhibited (Fig. 4a and Additional file 4: Table S3). Similarly, genes in the disease group were significant highly enriched in two pathways, with 18 pathways enriched in the control group (p value < 0.05) according to the GSEA results (Fig. 4b and Additional file 4: Table S4). Seven pathways, including the ubiquitin-mediated proteolysis pathway, primary immunodeficiency pathway, SLE pathway, lysosome pathway, glycosaminoglycan degradation pathway, complement and coagulation cascades pathway, and the arrhythmogenic right ventricular cardiomyopathy (ARVC) pathway overlapped in both the GSVA and GSEA results, and are listed in Table 2. Furthermore, only two pathways (the complement and coagulation cascades pathway and the SLE pathway) in the KEGG pathway enrichment results of dataset E-MTAB-69 DEGs were overlapped with intersection of the GSVA and GSEA results.

**Table 2:** The pathways overlapped in results of GSVA and GSEA

Pathway	GSVA		GSEA	
	logFC	Adj. P. val	NES	p-val
KEGG_UBIQUITIN_MEDIATED_PROTEOLYSIS	0.257353	0.000123	1.601199	0.018443
KEGG_PRIMARY_IMMUNODEFICIENCY	0.256664	0.014024	1.54737	0.032389
KEGG_SYSTEMIC_LUPUS_ERYTHEMATOSUS	− 0.30003	0.014921	− 1.46851	0.039293
KEGG_LYSOSOME	− 0.26996	0.00536	− 1.5659	0.038536
KEGG_GLYCOSAMINOGLYCAN_ DEGRADATION	− 0.32315	0.000742	− 1.68337	0.005941
KEGG_COMPLEMENT_AND_COAGULATION_ CASCADES	− 0.36335	1.77E−06	− 1.54136	0.005917
KEGG_ARRHYTHMOGENIC_RIGHT_ VENTRICULAR_CARDIOMYOPATHY_ARVC	− 0.20114	0.003886	− 1.47921	0.008163

*NES* Normalized Enrichment Score

### Immune cell type pattern alteration in MS cases

**Fig. 5:**
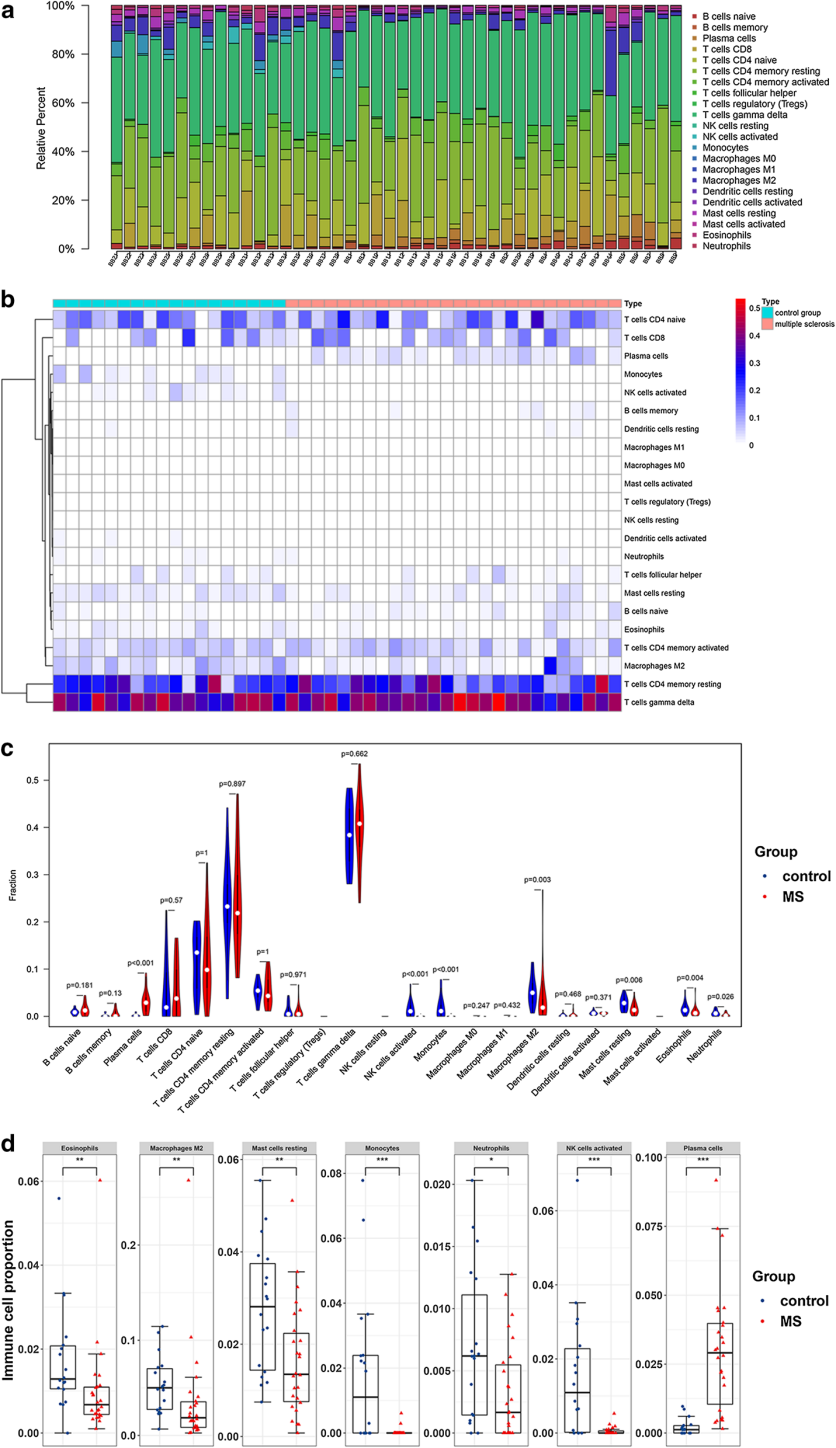
The landscape of immune cell distributed pattern in MS and control groups of dataset E-MTAB-69. **a** Histogram of the fraction of 22 kinds of immune cell proportions in MS and control groups. X axis: each E-MTAB-69 sample; Y axis: percentage of each kind of immune cells. **b** Heatmap of 22 immune cell proportions in MS and control groups. **c** Violin plot shows the differences of 22 immune cell proportions between two groups. Red color represents MS cases, blue color represents controls. **d** Boxplot of comparisons of significantly altered immune cell proportion between two groups. Red color represents MS cases, blue color represents controls. *P < 0.05, **P < 0.01, ***P < 0.001 compared to the control group

The histogram shows the general distribution of various immune cells in each sample of dataset E-MTAB-69 (Fig. 5a). Different colors represent different types of immune cells. The height of each color represents the percentage of such cells in the sample, and the sum of the percentage of various immune cells equals 1. It was observed that the main infiltrating cells were: T cells gamma delta, T cells CD4 memory resting, T cells CD4 naïve, T cells CD8, T cells CD4 memory activated, and M2 Macrophages. Due to the limitations of the CIBERSORT algorithm, the distribution of some immune-cell subsets with low abundance expression in CSF samples of MS was not fully revealed. The proportion of immune cells in the comparison samples of the two groups showed individual differences. Cluster analysis of infiltrating immune cells in the disease and control data was an important means of determining the pathological processes and immune regulation mechanisms, as is shown in Fig. 5b. MS samples generally contained a lower proportion of eosinophils (p = 0.004), macrophages M2 (p = 0.003), resting mast cells (p = 0.006), monocytes (p < 0.001), neutrophils (p = 0.026), activated NK cells (p < 0.001) than control samples; whereas the plasma cells (p < 0.001) fraction was relatively higher (Fig. 5c). The boxplot (Fig. 5d) shows the details of the significantly altered immune cell proportion between the MS and control groups.

### Validation of CNS immune microenvironment alteration

**Fig. 6:**
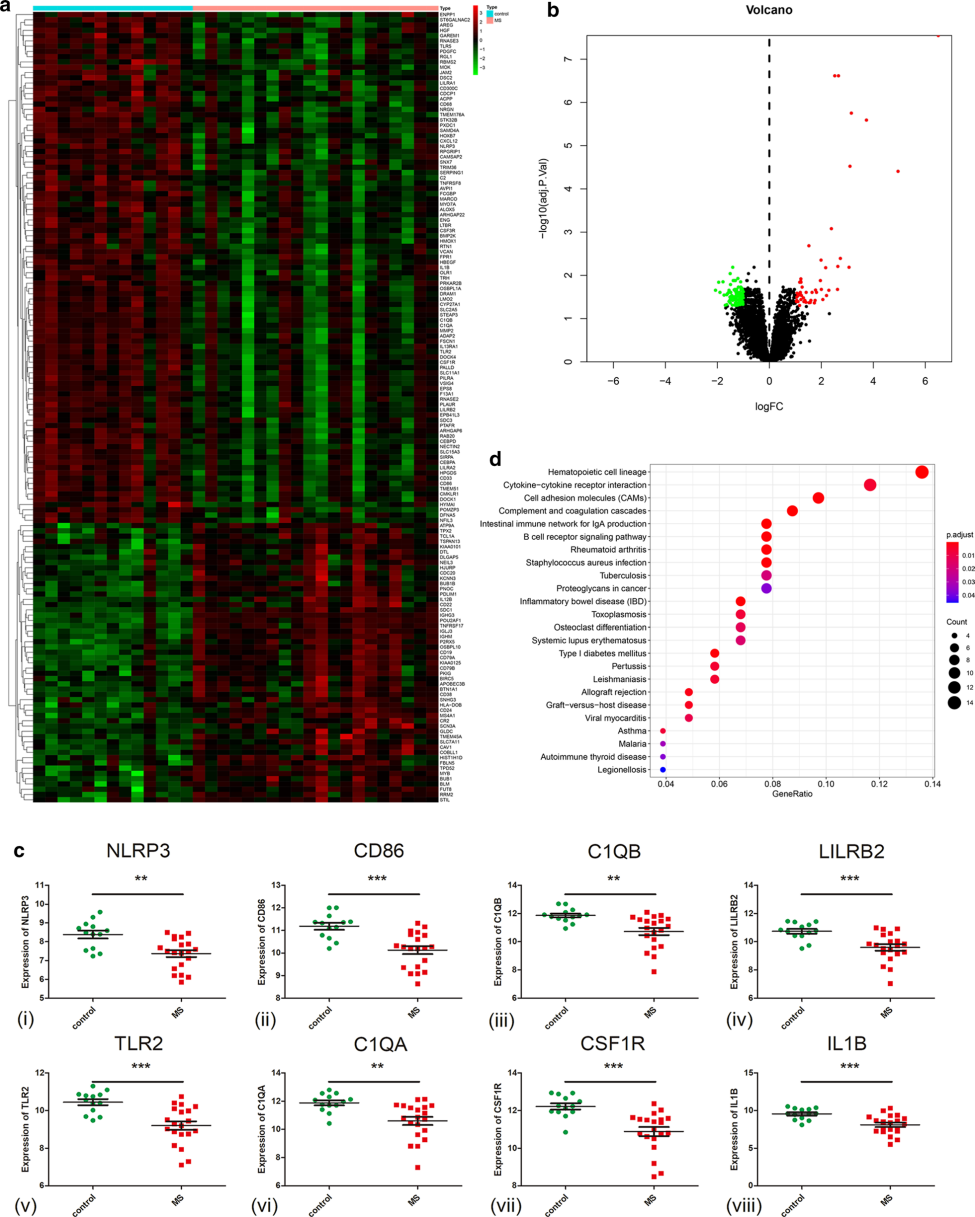
Differentially expressed genes of validation dataset E-MTAB-2374. **a** Heatmap of 150 DEGs between MS cases and control group. Green represents relatively downregulated genes, red represents upregulated genes, black represents genes showing no significant alteration. DEGs, differentially expressed genes. **b** Volcano plot showed 150 DEGs between MS cases and control group. **c** The expression of 8 overlapped core genes in validation dataset. **d** Bubble plot of KEGG gene set enrichment analysis of among all the DEGs. Gene ratio: the ratio of the enriched genes to the total number of genes in the relative pathway in the database. Count: the DEGs number enriched in each pathway

In Fig. 6a, the hierarchical cluster analysis heatmap showed significantly different distributions of gene expression patterns between the MS cases and control samples of the validation cohort. Under the threshold of FDR < 0.05 and |log2 (Fold Change)|> 1, a total of 150 DEGs (53 up-regulated and 97 down-regulated) were chosen for further analysis (Fig. 6b and Additional file 4: Table S5). Then, we found that eight genes were overlapped in the top 20 hub genes of the derivation cohort and 150 DEGs of the validation cohort. The expression details are listed in Table 3. These eight genes were recognized as core genes involved in MS disease. As shown in Fig. 6c(i–-(viii), we also found that NLRP3, LILRB2 C1QB, CD86, C1QA, CSF1R, IL1B and TLR2 were downregulated in the MS samples. The scatter plots of eight genes were consistent with the derivation cohort analysis results. The KEGG pathways of DEGs are shown in Fig. 6d, which were mainly enriched in immune-associated pathways, such as hematopoietic cell lineage, cytokine-cytokine receptor interaction, cell adhesion molecules, complement and coagulation cascades, intestinal immune network for IgA production, B cell receptor signaling pathway, SLE pathway and so on. In addition, we found that two pathways, the complement and coagulation cascades pathway and the SLE pathway, were overlapped in these KEGG results and in the former functional enrichment analysis such as in the GSEA, GSVA and KEGG results of the derivation cohort.

**Table 3:** The expression of the 8 genes overlapped in top 20 hub genes of derivation cohort and 150 DEGs of validation cohort

Gene symbol	logFC	t	P.Value	adj. P.Val	Up/down-regulated
NLRP3	− 1.015208	− 3.717949	0.0007216	0.0330639	Down-regulated
CD86	− 1.058162	− 4.334	0.0001233	0.0212309	Down-regulated
C1QB	− 1.146578	− 3.513766	0.0012729	0.0415789	Down-regulated
LILRB2	− 1.147991	− 3.781941	0.0006028	0.0308922	Down-regulated
TLR2	− 1.245801	− 4.140113	0.0002166	0.024029	Down-regulated
C1QA	− 1.285648	− 3.447825	0.0015253	0.0448478	Down-regulated
CSF1R	− 1.331567	− 4.240982	0.0001617	0.0222183	Down-regulated
IL1B	− 1.447641	− 3.834274	0.0005199	0.0292885	Down-regulated

*Down-regulated* down-regulated in MS cases

**Fig. 7:**
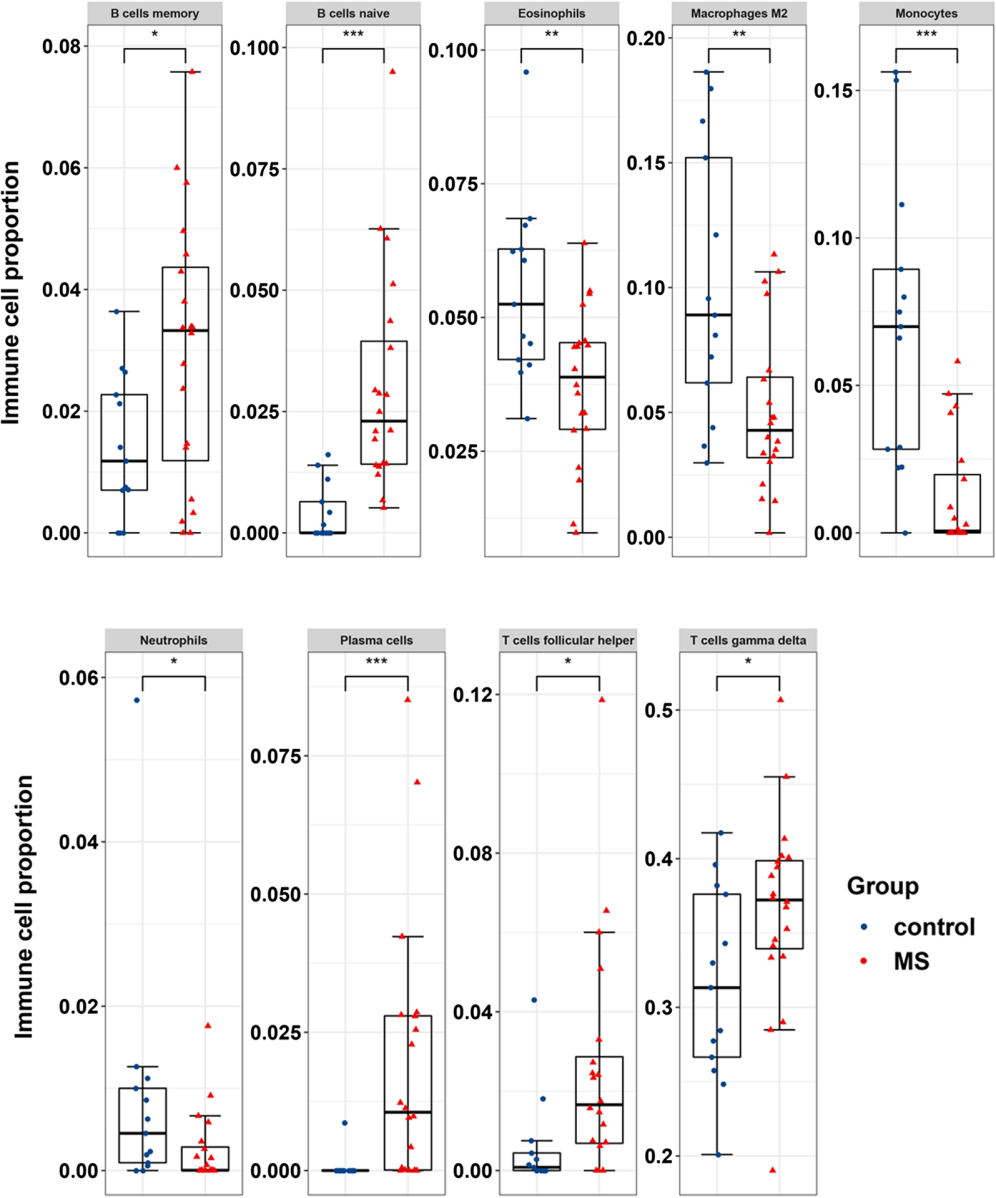
Boxplot of comparisons of significantly altered immune cell proportion between MS and control groups in validation dataset E-MTAB-2374. Red color represents MS cases, blue color represents control group. *P < 0.05, **P < 0.01, ***P < 0.001 compared to the control group

We found that MS samples generally contained a lower proportion of eosinophils (p = 0.007), macrophages M2 (p = 0.009), monocytes (p < 0.001), neutrophils (p = 0.027) than the control samples; whereas the plasma cells (p < 0.001), B memory cells (p = 0.034), naive B cells (p < 0.001), follicular helper T cells (p = 0.012) and gamma delta T cells (p = 0.04) fractions were relatively higher (Fig. 7 and Additional file 1: Figure S1). Five types of immune cells (plasma cells, monocytes, M2 macrophages, neutrophils and eosinophils) in the cerebrospinal fluid (CSF) were shown to be significantly different between the MS and control groups.

Furthermore, the combined ImmuCellAI online analysis results of dataset E-MTAB-69 (as shown in Additional file 2: Figure S2) and E-MTAB-2374 (as shown in Additional file 3: Figure S3) indicated that CD8 + naïve T cell, Th17, effector memory T (Tem) cell, mucosal-associated invariant T (MAIT) cell, dendritic cell (DC), B cell, Monocyte, Macrophage and Neutrophil had abundance differences between MS and control groups. This online tool is mainly for 18 T cell subtype and 6 other immunocytes abundance prediction, so its immune cell classification is not one-to-one correspondence with our CIBERSORT analysis. The results from these two methods roughly matched, however, were still needed further experimental verification in the future.

## Discussion

MS is an autoimmune disease characterized by demyelination of the CNS and infiltration of inflammatory cells. The condition relapses and progresses, often leading to lifelong disabilities. During the pathogenesis of multiple sclerosis, a variety of immune-related molecules and pathways are altered. We identified eight core molecules in our findings (NLRP3, LILRB2, C1QB, CD86, C1QA, CSF1R, IL1B and TLR2), all of which, with the exception of C1QB, have previously been reported to be associated with MS or EAE. However, previous research has focused on the protein level alteration in the CNS tissue or supernatant of CSF [26–32].

Recently, Hammond and colleagues [26] found that complement C1q A chain (C1qA) mRNA expression, and C1q protein expression, were both significantly increased in the hippocampus of EAE mice compared to control groups. Expression of the transcript for C1qA was noted in the neurons in the MS cortical and deep grey matter [33]. The variant rs158772 of C1QA was associated with a 71% increase in risk of sustained low-contrast letter acuity loss, which indicated visual system degeneration in MS [34]. Previous studies have reported that neurological damage and degenerative changes could influence the expression of complement C1q B chain (C1qB). Experimental lesions (kainic acid-induced) in the hippocampus and in other brain regions increased C1qB mRNA [35]. Grewal and colleagues found that C1qB mRNA increases in association with neurodegeneration in sporadic amyotrophic lateral sclerosis (ALS) [36]. In addition, C1q deficiency caused by the splicing mutation in the C1qB gene is closely correlated with the development of SLE [37, 38].

Furthermore, an important protein of the innate immune system, nucleotide-binding leucine-rich repeat family pyrin domain containing 3 (NLRP3) has been reported to mediate pyroptosis, and to be associated with various autoimmune disorders such as neuromyelitis optica spectrum disorder (NMOSD) and MS [39]. Recently, our team [27] as well as many other researchers [28, 29] have found that the NLRP3-mediated innate immune pathways may be a novel target for future treatments for MS. As a consequence of NLRP3 inflammasome activation, the expression of inflammatory genes IL1B increased in CNS tissues taken from cases with MS and from animal models with EAE [40]. Another important study demonstrated that patients with high IL1B gene levels progressed significantly faster compared to primary progressive multiple sclerosis (PPMS) patients with low IL1B expression levels in peripheral blood mononuclear cells (PBMCs), which indicated that IL1B could be a prognostic biomarker in patients with PPMS [41].

Using quantitative RT-PCR, leukocyte immunoglobulin-like receptor B2 (LILRB2), also called immunoglobulin-like transcript 4 (ILT4), has been reported to be upregulated in active lesions in the MS brain compared to the control brain [42]. In addition, LILRB2 was also induced in monocytes by IFN beta treatment in vitro, and led to a beneficial effect in MS.

Recently, Hagan and colleagues found that colony-stimulating factor-1 receptor (CSF1R) was elevated in the CNS tissue of MS cases with progressive disease [30]. Furthermore, their research demonstrated that CSF1R inhibition could reduce harmful microglial proliferation, modulate microglial phenotypes and reduce subsequent demyelination and neurodegeneration. CSF1R gene mutations were reported to be associated with hereditary diffuse leukoencephalopathy with spheroids (HDLS) which led to demyelination and axonal degeneration with spheroids of the CNS [43, 44], presenting as primary progressive MS.

It is known that the expression of Toll-like receptor 2 (TLR2) is increased in CNS tissues [31], and even in infiltrated inflammatory cells in the CNS [45]) taken from cases with MS and EAE animal models, as well as in the peripheral blood mononuclear cells (PBMCs) [46]. Enhanced TLR2 responsiveness plays a critical role in the pathogenesis of MS [47], and TLR2 could inhibit the maturation and remyelination of oligodendrocyte precursor cells [48]. Reducing innate immune signals by inducing TLR2 tolerance may be a novel approach to alleviating inflammation and repairing myelin sheaths in MS [49].

The CD86 molecule (also known as B7-2) is expressed both in MS lesions and inflammatory infarcts, mainly on macrophages [32]. A study found that CD86 mRNA in the CSF cells of MS cases showed no significant difference from that of a control group [50]. However, another study reported that the costimulatory molecule CD86 expressed by T cells in CSF was low in patients with MS compared to noninflammatory control subjects [51], which is a similar finding to ours. Moreover, this study focused on transcriptional level analysis of the CSF cells of both MS cases and control groups in order to find a missing part to complete the picture of the immune microenvironment. CSF cell counts are low, especially in individuals without sustained inflammation, which may account for why CSF cell gene expression levels are rarely reported.

It is of interest that these eight genes were downregulated in the CSF cells, which was in contrast to the gene expression trends in CSF supernatant and in lesions. We postulated that the gene expression changes in CSF cells are due to the negative feedback regulation of the immune microenvironment, and occur in order to maintain homeostasis. Furthermore, we hypothesized that these molecular changes may be related to protein degradation and activation of the ubiquitin–proteasome pathway, which is consistent with our GSVA and GSEA findings (Fig. 4 and Table 2). The cells in CSF, disease lesions, and the CSF supernatant which bathed the CNS tissue together constitute the immune microenvironment of the disease. As the Chinese saying goes, ‘pull one hair, and the whole body moves’; in other words, a slight change will affect everything else. Our study suggests that the expression of related molecules in the cells of the CSF also changes according to the compositional changes of the CSF. Furthermore, our results are consistent with the findings of the original microchip research [22], such as the findings that B cell maturation factor TNF receptor superfamily member 17 (TNFRSF17), and POU class 2 homeobox associating factor 1 (POU2AF1) which is involved in Ig gene transcription, were highly expressed in MS, while AIF1 was down expressed in MS (see Additional file 4: Table S2 for details). Previous studies have indicated that POU2AF1 is a B-cell-specific transcriptional co-activator, which directly bound to TNFRSF17 and enhance its transcription [52], and AIF1 could induce a M2-like phenotype of macrophages [53]. Our results indicated that TNFRSF17, POU2AF1 upregulated and plasma cells increased in MS cases, AIF1 downregulated and M2 macrophages decreased in MS cases. The trend of gene expression and the trend of cell proportion were mutually verified, which also coincided with achievements of predecessors [52, 53] on this point and showed credibility of the method we used. The findings from the E-MTAB-69 dataset will be intersected with those from E-MTAB-2374, which is equivalent to further expanding the sample size and searching for common differentially expressed genes, which is different from the focus of the original microchip research.

Our study demonstrated that the complement and coagulation cascades pathway and the SLE pathway were dysregulated in MS cases [22]. MS and SLE are common autoimmune diseases and may have a shared pathogenesis. Previous research [54] found that the occurrence and development of these two autoimmune diseases may be associated with lysosomes and phagocytosis, which leads to abnormal immune-related reactions and hence causes disease. Moreover, in 2019, Magliozzi and colleagues found that intrathecal dysregulation of complement and coagulation cascade pathways, as well as B-cell and monocyte activity, are strictly correlated with cortical damage at the early stages of MS [55]. Indeed, this field is the subject of increasing attention from researchers. Koudriavtseva and colleagues have undertaken a multi-center, prospective, controlled study to determine the exact links between activation of the coagulation/complement system and cerebral hypoperfusion in RRMS cases [56]. It has been suggested that interfering with the coagulation system might provide a novel therapeutic target in the treatment of MS and demyelinating diseases.

In this study, we used the CIBERSORT algorithm to analyze gene expression data to determine the immune cells ratio alteration in MS. We found that, in the CSF of MS cases, plasma cells increased, and monocytes decreased [22], a finding which corresponded with the speculative results of original microchip research. However, changes in the ratio of M2 macrophages, eosinophils and neutrophils were not mentioned by them and worthy of further experimental research.

The immune cells and immune reactions play a vital role in MS progress. The CIBERSORT analysis could convert the expression matrix into the immunocytes fraction matrix, which is helpful for better understanding the pathological process of diseases, especially immune-related diseases. Single-cell transcriptomics is an emerging technology which could elucidate the heterogeneity of complex tissues. Single-cell analysis of cells in CSF could help us to discover new and unknown populations of cells [41]. However, because of its high cost, single cell analysis technology has so far not been widely used, and its clinical application is limited. The accuracy of CIBERSORT has been validated by fluorescence activated cell sorting (FACS) technique, and before single-cell sequencing is widely available, CIBERSORT will be a simple and effective method to investigate immunocyte pattern of CSF in MS.

Although we have found and verified our research results with two independent datasets, we have to admit that there are some limitations in current study. First, this study is based on two public datasets uploaded some years ago, of which the complete follow-up information of clinical samples is lacked. Second, a patient’s first clinical episode of neurological symptoms is often diagnosed as clinical isolated syndrome (CIS), and at the initial diagnosis, testing for oligoclonal bands and other demyelinating related markers is undertaken to differentiate the patient from Neuromyelitis optica (NMO) or other diseases. The diagnosis of MS is confirmed by the recurrent nature of the disease, with the extended course of treatment determined by imaging results and clinical symptoms. Therefore, there are some limitations to simply obtaining clinical CSF samples. Finally, our findings based on retrospective bioinformatics analysis should be verified by following up CIS patients, cell and animal experiments in the future.

## Conclusions

In summary, our study is the first to use the CIBERSORT method to analyze the immune cell subtypes distribution pattern in CSF samples of MS. Our study attempted to better understand the alteration of the microenvironment in, and the cause of, MS. More in-depth research of these core genes, pathways and differential immune cells may further uncover the underlying mechanisms and pathological process of MS.

## Supplementary Information

**Additional file 1: Figure S1.** Violin plot of significant differential immune cells in MS and control groups. The control group is shown in blue and MS group is shown in red.

**Additional file 2: Figure S2.** The abundance differences of immune cells between MS and control groups in dataset E-MTAB-69 by the application of ImmuCellAI. 24 immune cell types including 18 T-cell subsets and 6 other important immune cells: CD4 + naïve cell, CD8 + naïve cell, cytotoxic T (Tc) cell, exhausted T (Tex) cell, type 1 regulatory T (Tr1) cell, natural regulatory T (nTreg) cell, induced regulatory T (iTreg) cell, Th1, Th2, Th17, T follicular helper (Tfh) cell, central memory T (Tcm) cell, effector memory T (Tem) cell, natural killer T (NKT) cell, mucosal-associated invariant T (MAIT) cell, gamma delta (γδ) T (Tgd) cell, CD4 + T cell, CD8 + T cell, dendritic cell (DC), B cell, monocyte, macrophage, natural killer (NK) cell and neutrophil. A p value < 0.05 was considered to indicate a statistically significant difference. Red color represents MS case, blue color represents control group.

**Additional file 3: Figure S3.** The abundance differences of immune cells between MS and control groups in dataset E-MTAB-2374 by the application of ImmuCellAI. A p value < 0.05 was considered to indicate a statistically significant difference. Red color represents MS case, blue color represents control group.

**Additional file 4: Table S1.** The information of control samples in these two datasets. **Table S2.** The differentially expressed genes of dataset E-MTAB-69. **Table S3.** GSVA results of the KEGG gene-set enriched in samples of derivation dataset (MS Vs Control). **Table S4.** GSEA results of the most of the significantly altered pathways were activated in the derivation dataset. **Table S5.** The differentially expressed genes of dataset E-MTAB-2374.

## Data Availability

The data used to support the findings of this study are available from the corresponding author upon request.

## Abbreviations

BBB: Blood–brain barrier
CIBERSORT: Cell-type identification by estimating relative subsets of RNA transcripts
CIS: Clinical isolation syndrome
CNS: Central nervous system
CSF: Cerebrospinal fluid
EAE: Experimental autoimmune encephalomyelitis
FACS: Fluorescence activated cell sorting
GO: Gene Ontology
GSEA: Gene set enrichment analysis
GSVA: Gene set variation analysis
HDLS: Hereditary diffuse leukoencephalopathy with spheroids
KEGG: Kyoto Encyclopedia of Genes and Genomes
MBP: Myelin basic protein
MOG: Myelin oligodendrocyte glycoprotein
MS: Multiple sclerosis
NES: Normalized Enrichment Score
OCB: Oligoclonal bands
SLE: Systemic lupus erythematosus
TLR2: Toll-like receptor 2
