# Supplementary figures and images for: Cerebrospinal fluid cells immune landscape in multiple sclerosis
Source: J Transl Med. 2021 Mar 25;19:125. doi: 10.1186/s12967-021-02804-7 (PMC7995713; doi:10.1186/s12967-021-02804-7)

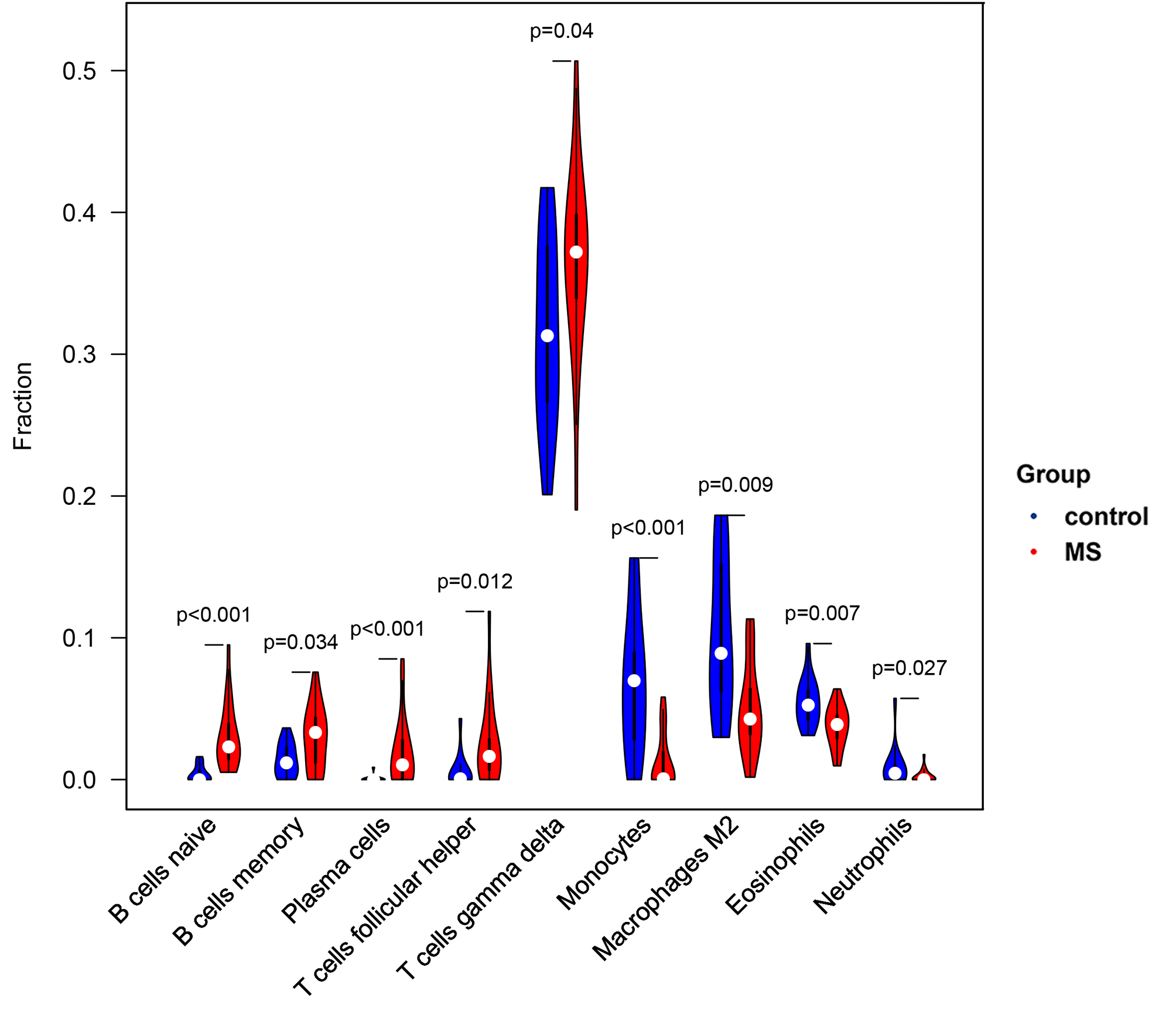

Supplement: Supplementary file 1 — Additional file 1: Figure S1. Violin plot of significant differential immune cells in MS and control groups. The control group is shown in blue and MS group is shown in red. [file 12967_2021_2804_MOESM1_ESM.tif]

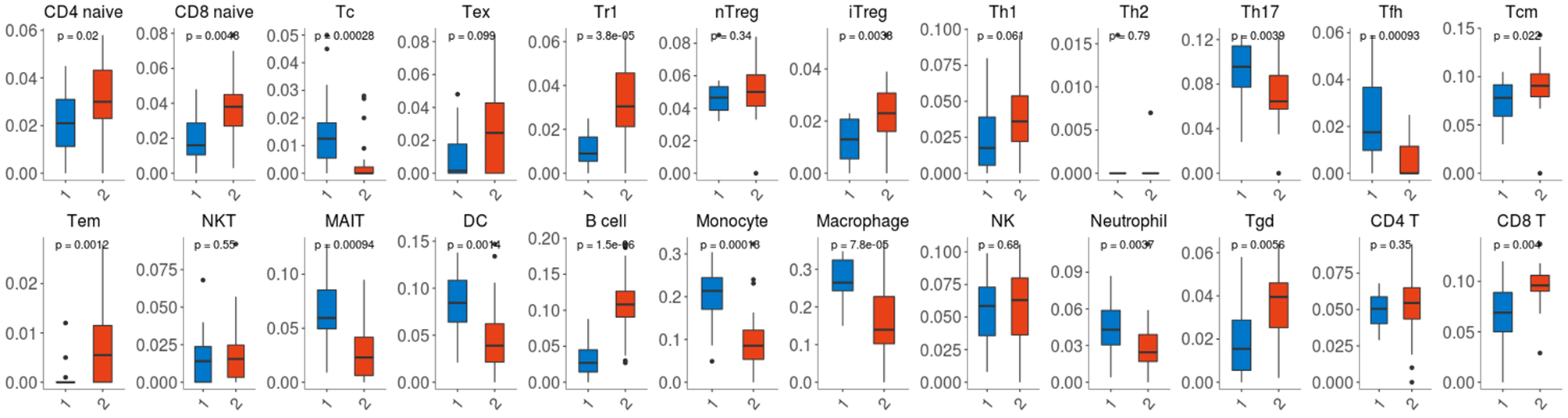

Supplement: Supplementary file 2 — Additional file 2: Figure S2. The abundance differences of immune cells between MS and control groups in dataset E-MTAB-69 by the application of ImmuCellAI. 24 immune cell types including 18 T-cell subsets and 6 other important immune cells: CD4 + naïve cell, CD8 + naïve cell, cytotoxic T (Tc) cell, exhausted T (Tex) cell, type 1 regulatory T (Tr1) cell, natural regulatory T (nTreg) cell, induced regulatory T (iTreg) cell, Th1, Th2, Th17, T follicular helper (Tfh) cell, central memory T (Tcm) cell, effector memory T (Tem) cell, natural killer T (NKT) cell, mucosal-associated invariant T (MAIT) cell, gamma delta (γδ) T (Tgd) cell, CD4 + T cell, CD8 + T cell, dendritic cell (DC), B cell, monocyte, macrophage, natural killer (NK) cell and neutrophil. A p value < 0.05 was considered to indicate a statistically significant difference. Red color represents MS case, blue color represents control group. [file 12967_2021_2804_MOESM2_ESM.tif]

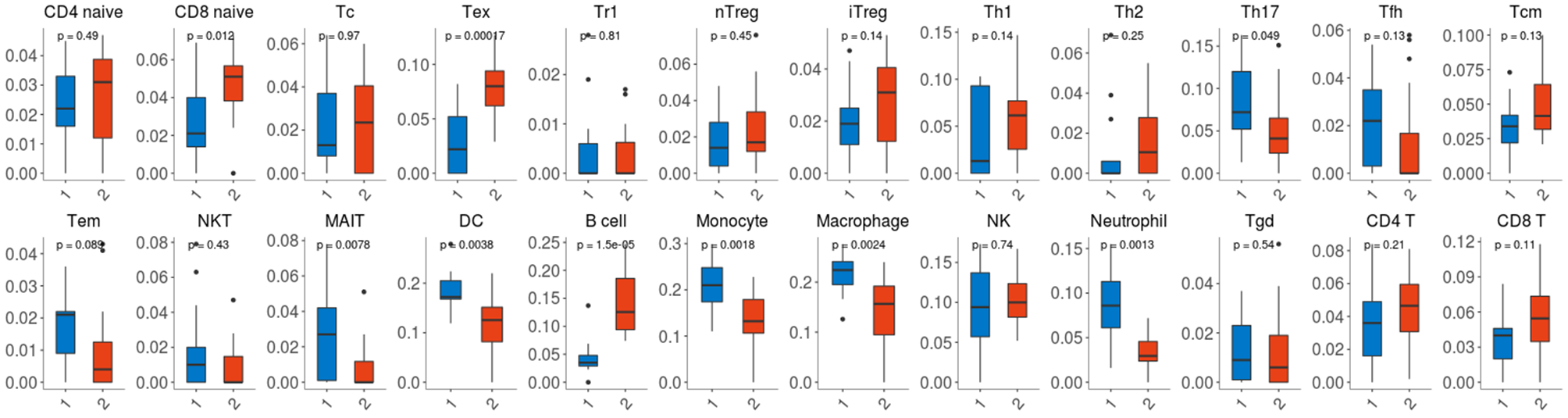

Supplement: Supplementary file 3 — Additional file 3: Figure S3. The abundance differences of immune cells between MS and control groups in dataset E-MTAB-2374 by the application of ImmuCellAI. A p value < 0.05 was considered to indicate a statistically significant difference. Red color represents MS case, blue color represents control group. [file 12967_2021_2804_MOESM3_ESM.tif]
