# Supplementary material for: Cerebrospinal fluid cells immune landscape in multiple sclerosis
Source: J Transl Med. 2021 Mar 25;19:125. doi: 10.1186/s12967-021-02804-7 (PMC7995713; doi:10.1186/s12967-021-02804-7)
Supplement: Supplementary file 4 — Additional file 4: Table S1. The information of control samples in these two datasets. Table S2. The differentially expressed genes of dataset E-MTAB-69. Table S3. GSVA results of the KEGG gene-set enriched in samples of derivation dataset (MS Vs Control). Table S4. GSEA results of the most of the significantly altered pathways were activated in the derivation dataset. Table S5. The differentially expressed genes of dataset E-MTAB-2374. [file 12967_2021_2804_MOESM4_ESM.doc]

| **Supplementary Table 1.** The information of MS and control samples in two datasets. | | |
| --- | --- | --- |
| dataset | detailed information of clinical samples | number |
| **E-MTAB-69** | |  |
| control samples with other non-inflammatory neurological diseases  (N=18) | psychosis | 4 |
|  | vertigo | 2 |
|  | paresthesia | 2 |
|  | cervical spinal stenosis | 1 |
|  | ependymoma | 1 |
|  | headache | 1 |
|  | hereditary spastic paresis | 1 |
|  | migraine | 1 |
|  | musclepain | 1 |
|  | neurasthenia | 1 |
|  | sensory symptoms | 1 |
|  | vertebral dissection | 1 |
|  | dissociative motor disorder | 1 |
| MS cases  (N=26) | sampled during a disease bout initiated no more than 2 weeks earlier | 12 |
|  | sampled during remission, at least 6 months after an earlier relapse | 14 |
| **E-MTAB-2374** | |  |
| control samples with other neurological diseases  (N=13) | sarcoidosis | 1 |
|  | HTLV 1 myelopathy | 1 |
|  | movement disorder (Parkinson’s disease) | 1 |
|  | cerebral vascular disease (stroke) | 2 |
|  | spinal stenosis | 1 |
|  | lymphoma and ischemic myelopathy (unknown, but not multiple sclerosis) | 7 |
| MS cases  (N=20) | relapsing-remitting MS | 5 |
|  | primary progressive MS | 6 |
|  | secondary progressive MS | 9 |
|  | active disease within 6 months prior to the CSF sample collection | |
|  |  |  |

| **Supplementary Table 2.** The differentially expressed genes of dataset E-MTAB-69. | | | | | | |
| --- | --- | --- | --- | --- | --- | --- |
| Gene ID | logFC | AveExpr | t | P.Value | adj.P.Val | up/down-regulated |
| IGHG3 | 6.7004387 | 9.2289319 | 9.3553172 | 3.72E-12 | 3.77E-08 | upregulated |
| NLRP3 | -1.127687 | 6.1454065 | -7.976743 | 3.53E-10 | 8.93E-07 | down-regulated |
| TNFRSF17 | 3.3585915 | 6.147423 | 7.5079655 | 1.72E-09 | 3.12E-06 | upregulated |
| IGHM | 2.4938451 | 6.7866061 | 6.8857278 | 1.45E-08 | 9.16E-06 | upregulated |
| UBE2J1 | 1.1632988 | 8.2741288 | 6.7345613 | 2.43E-08 | 1.26E-05 | upregulated |
| IGLJ3 | 2.2665716 | 6.9646862 | 6.617465 | 3.64E-08 | 1.75E-05 | upregulated |
| IER3 | -1.794899 | 9.3953723 | -6.544798 | 4.67E-08 | 2.06E-05 | down-regulated |
| PLAU | -1.018345 | 6.1980339 | -6.500062 | 5.45E-08 | 2.30E-05 | down-regulated |
| KIAA0125 | 1.9429686 | 6.5568002 | 6.4567416 | 6.32E-08 | 2.32E-05 | upregulated |
| NR4A2 | -1.404282 | 10.015886 | -6.440021 | 6.70E-08 | 2.34E-05 | down-regulated |
| POU2AF1 | 3.0505602 | 8.263533 | 6.430047 | 6.93E-08 | 2.34E-05 | upregulated |
| ALDH2 | -1.200852 | 8.5813666 | -6.397585 | 7.75E-08 | 2.53E-05 | down-regulated |
| RXRA | -1.072641 | 8.4593116 | -6.296076 | 1.10E-07 | 3.20E-05 | down-regulated |
| RAB20 | -1.49258 | 8.1353025 | -6.0852 | 2.27E-07 | 4.92E-05 | down-regulated |
| TNFSF13 | -1.00174 | 6.6839141 | -6.083103 | 2.28E-07 | 4.92E-05 | down-regulated |
| MS4A1 | 1.4470139 | 5.9520378 | 6.0386781 | 2.66E-07 | 5.50E-05 | upregulated |
| CAV1 | 1.2259306 | 5.2743968 | 5.9890245 | 3.15E-07 | 5.92E-05 | upregulated |
| CD38 | 1.773298 | 6.6501327 | 5.8843283 | 4.52E-07 | 7.29E-05 | upregulated |
| AIF1 | -1.043776 | 7.2929698 | -5.837666 | 5.30E-07 | 7.89E-05 | down-regulated |
| PID1 | -1.543674 | 6.5021937 | -5.785041 | 6.34E-07 | 8.92E-05 | down-regulated |
| ADAP2 | -1.364718 | 7.804713 | -5.707701 | 8.26E-07 | 0.0001116 | down-regulated |
| PXDC1 | -1.108911 | 7.582402 | -5.696373 | 8.59E-07 | 0.0001129 | down-regulated |
| CD83 | -1.187361 | 9.9435127 | -5.646748 | 1.02E-06 | 0.0001272 | down-regulated |
| ARMCX1 | -1.264303 | 6.1703359 | -5.53762 | 1.48E-06 | 0.0001559 | down-regulated |
| PFKFB3 | -1.016288 | 9.1909896 | -5.537164 | 1.48E-06 | 0.0001559 | down-regulated |
| NR4A3 | -1.027115 | 6.1072894 | -5.498605 | 1.68E-06 | 0.0001706 | down-regulated |
| IRS2 | -1.013646 | 9.9138145 | -5.495606 | 1.70E-06 | 0.0001706 | down-regulated |
| A2M | -1.920534 | 8.8416205 | -5.492704 | 1.72E-06 | 0.0001706 | down-regulated |
| IGSF6 | -1.289548 | 6.7395 | -5.479428 | 1.80E-06 | 0.0001718 | down-regulated |
| DSE | -1.157983 | 9.1462638 | -5.470485 | 1.85E-06 | 0.0001725 | down-regulated |
| CYFIP1 | -1.339799 | 10.238845 | -5.421105 | 2.19E-06 | 0.0001926 | down-regulated |
| C3 | -1.818398 | 8.7555311 | -5.390705 | 2.43E-06 | 0.0002044 | down-regulated |
| CH25H | -2.406343 | 7.6331741 | -5.386736 | 2.46E-06 | 0.0002044 | down-regulated |
| PTGS1 | -1.038153 | 6.4070127 | -5.380084 | 2.52E-06 | 0.0002073 | down-regulated |
| PLXNB2 | -1.002906 | 7.1704871 | -5.356686 | 2.73E-06 | 0.0002191 | down-regulated |
| SGK1 | -1.319083 | 10.6877 | -5.338816 | 2.90E-06 | 0.0002309 | down-regulated |
| STEAP3 | -1.127652 | 6.4899098 | -5.327244 | 3.01E-06 | 0.0002364 | down-regulated |
| P3H2 | -1.038691 | 6.4659586 | -5.312272 | 3.17E-06 | 0.0002412 | down-regulated |
| ADGRE2 | -1.10526 | 5.7607821 | -5.265731 | 3.71E-06 | 0.0002682 | down-regulated |
| EGR2 | -1.23895 | 7.6951459 | -5.229394 | 4.19E-06 | 0.0002867 | down-regulated |
| CDK2AP1 | -1.051194 | 10.066761 | -5.17422 | 5.05E-06 | 0.0003154 | down-regulated |
| TGFBI | -1.393946 | 10.088957 | -5.167152 | 5.17E-06 | 0.0003184 | down-regulated |
| IL1B | -1.353393 | 7.2830393 | -5.158775 | 5.32E-06 | 0.0003223 | down-regulated |
| CEBPD | -1.376592 | 9.146085 | -5.143326 | 5.60E-06 | 0.0003354 | down-regulated |
| HLA-DMB | -1.040381 | 10.061925 | -5.129602 | 5.86E-06 | 0.0003451 | down-regulated |
| PTGS2 | -1.585185 | 6.5826698 | -5.124249 | 5.97E-06 | 0.0003489 | down-regulated |
| CTNND1 | -1.174755 | 7.6475522 | -5.114263 | 6.17E-06 | 0.0003551 | down-regulated |
| KIF11 | 1.1225483 | 5.3709085 | 5.0931161 | 6.63E-06 | 0.0003686 | upregulated |
| CFD | -1.568775 | 7.2026742 | -5.085396 | 6.80E-06 | 0.0003722 | down-regulated |
| OLR1 | -2.007576 | 5.4999452 | -5.082052 | 6.88E-06 | 0.0003723 | down-regulated |
| FCGR2A | -1.580725 | 8.3382315 | -5.045631 | 7.77E-06 | 0.0004001 | down-regulated |
| SNX7 | -1.619275 | 5.50252 | -5.035321 | 8.04E-06 | 0.0004051 | down-regulated |
| RIN2 | -2.047304 | 8.0019473 | -5.033614 | 8.09E-06 | 0.0004054 | down-regulated |
| DOCK4 | -1.966811 | 7.0872171 | -5.02473 | 8.33E-06 | 0.0004135 | down-regulated |
| HBEGF | -1.206821 | 7.4236064 | -5.001234 | 9.01E-06 | 0.00043 | down-regulated |
| APOBEC3B | 1.6117339 | 5.9581096 | 5.0002318 | 9.04E-06 | 0.00043 | upregulated |
| ITGB5 | -1.455511 | 8.9839543 | -4.998516 | 9.09E-06 | 0.00043 | down-regulated |
| CAMSAP2 | -1.017141 | 6.5447539 | -4.997421 | 9.13E-06 | 0.00043 | down-regulated |
| STAB1 | -1.416853 | 6.7210821 | -4.991792 | 9.30E-06 | 0.000432 | down-regulated |
| AVPI1 | -1.21427 | 6.8961164 | -4.975764 | 9.81E-06 | 0.0004486 | down-regulated |
| MARCKS | -1.022881 | 7.1752782 | -4.927873 | 1.15E-05 | 0.0004917 | down-regulated |
| EPB41L2 | -1.351352 | 8.8158105 | -4.921501 | 1.18E-05 | 0.000498 | down-regulated |
| NAMPT | -1.086624 | 9.7773996 | -4.907796 | 1.23E-05 | 0.0005106 | down-regulated |
| ETS2 | -1.080873 | 7.059623 | -4.901952 | 1.25E-05 | 0.0005142 | down-regulated |
| IL13RA1 | -1.044601 | 7.8282581 | -4.883519 | 1.33E-05 | 0.0005359 | down-regulated |
| FCGR1B | -1.721089 | 7.0193903 | -4.837594 | 1.55E-05 | 0.0005957 | down-regulated |
| CXCL8 | -1.991659 | 8.4593359 | -4.817948 | 1.66E-05 | 0.0006239 | down-regulated |
| FCGBP | -1.719615 | 9.8374711 | -4.796399 | 1.78E-05 | 0.0006483 | down-regulated |
| F13A1 | -1.856283 | 8.1338308 | -4.771529 | 1.93E-05 | 0.0006866 | down-regulated |
| LMO2 | -1.190337 | 8.2936443 | -4.740672 | 2.14E-05 | 0.0007445 | down-regulated |
| EPB41L3 | -1.727133 | 6.8609847 | -4.738828 | 2.15E-05 | 0.0007458 | down-regulated |
| BUB1B | 1.2058294 | 6.9500335 | 4.7370112 | 2.17E-05 | 0.0007458 | upregulated |
| RRM2 | 2.1179146 | 6.9774964 | 4.7157706 | 2.32E-05 | 0.0007797 | upregulated |
| CD79A | 1.1958403 | 6.0458332 | 4.7147438 | 2.33E-05 | 0.0007797 | upregulated |
| FN1 | -1.534989 | 6.6706417 | -4.705012 | 2.41E-05 | 0.0007962 | down-regulated |
| LILRB2 | -1.260703 | 6.1891166 | -4.694971 | 2.49E-05 | 0.0008038 | down-regulated |
| RAB31 | -1.234518 | 8.8967189 | -4.642954 | 2.95E-05 | 0.0009024 | down-regulated |
| MNDA | -1.032417 | 8.8965392 | -4.618707 | 3.19E-05 | 0.0009521 | down-regulated |
| PNOC | 1.1183854 | 6.017002 | 4.608726 | 3.30E-05 | 0.0009719 | upregulated |
| CX3CR1 | -1.060807 | 10.875117 | -4.596228 | 3.44E-05 | 0.0009944 | down-regulated |
| TLR2 | -1.40272 | 8.8150891 | -4.593874 | 3.46E-05 | 0.0009964 | down-regulated |
| RAB13 | -1.331072 | 8.5052573 | -4.591298 | 3.49E-05 | 0.0010019 | down-regulated |
| EPS8 | -1.545755 | 7.8614619 | -4.515784 | 4.47E-05 | 0.0011905 | down-regulated |
| CSTA | -1.299843 | 9.6075549 | -4.503841 | 4.64E-05 | 0.0012278 | down-regulated |
| GGH | 1.0800691 | 6.0585625 | 4.4638276 | 5.29E-05 | 0.0013504 | upregulated |
| GATM | -1.462894 | 6.1464926 | -4.462277 | 5.31E-05 | 0.0013521 | down-regulated |
| CSF1R | -1.212137 | 7.614725 | -4.459877 | 5.36E-05 | 0.0013545 | down-regulated |
| TNS3 | -1.015121 | 7.8503817 | -4.45536 | 5.43E-05 | 0.0013622 | down-regulated |
| ATF3 | -1.073683 | 6.288154 | -4.410117 | 6.29E-05 | 0.0015312 | down-regulated |
| SPP1 | -2.278272 | 9.4834367 | -4.403481 | 6.43E-05 | 0.0015532 | down-regulated |
| VSIG4 | -1.454725 | 8.4809549 | -4.384442 | 6.83E-05 | 0.0016224 | down-regulated |
| C3AR1 | -1.333582 | 9.1867155 | -4.380978 | 6.91E-05 | 0.0016311 | down-regulated |
| CD14 | -1.899109 | 9.4825601 | -4.37631 | 7.01E-05 | 0.0016443 | down-regulated |
| DEPTOR | -1.205384 | 7.5469537 | -4.350072 | 7.63E-05 | 0.0017446 | down-regulated |
| CD93 | -1.154396 | 8.2846203 | -4.344644 | 7.77E-05 | 0.0017654 | down-regulated |
| HSPA6 | -1.202703 | 7.4973474 | -4.339645 | 7.89E-05 | 0.0017713 | down-regulated |
| TREM1 | -1.036859 | 5.9983705 | -4.322732 | 8.33E-05 | 0.0018302 | down-regulated |
| CD302 | -1.151877 | 8.8848629 | -4.308896 | 8.71E-05 | 0.0018846 | down-regulated |
| DLGAP5 | 1.2763406 | 4.7299106 | 4.2905399 | 9.24E-05 | 0.0019527 | upregulated |
| NUAK1 | -1.127016 | 5.7331608 | -4.281415 | 9.51E-05 | 0.0019735 | down-regulated |
| TRH | -1.01532 | 5.342223 | -4.275655 | 9.69E-05 | 0.0019938 | down-regulated |
| ADAM9 | -1.167201 | 7.9896234 | -4.263038 | 0.0001009 | 0.0020631 | down-regulated |
| PROS1 | -1.170547 | 5.6545922 | -4.177045 | 0.0001326 | 0.0024952 | down-regulated |
| CCL3 | -1.113449 | 7.3556567 | -4.17303 | 0.0001343 | 0.0025039 | down-regulated |
| FCER1A | -1.277188 | 10.338895 | -4.141677 | 0.0001483 | 0.0026858 | down-regulated |
| CXADR | -1.323597 | 6.7513962 | -4.130765 | 0.0001535 | 0.0027553 | down-regulated |
| HPGDS | -1.239525 | 6.1593862 | -4.120738 | 0.0001584 | 0.0027943 | down-regulated |
| CD86 | -1.086236 | 7.6627273 | -4.112828 | 0.0001624 | 0.0028352 | down-regulated |
| RNASE2 | -1.437384 | 7.2641008 | -4.104127 | 0.0001669 | 0.0028792 | down-regulated |
| NFIL3 | -1.014482 | 7.8787867 | -4.091985 | 0.0001734 | 0.0029511 | down-regulated |
| AIM2 | 1.004948 | 7.1862215 | 4.0702149 | 0.0001857 | 0.0030807 | upregulated |
| VCAN | -1.117669 | 6.4631916 | -4.066028 | 0.0001881 | 0.0031078 | down-regulated |
| AXL | -1.207978 | 8.2904952 | -4.057315 | 0.0001933 | 0.0031704 | down-regulated |
| SLC1A3 | -1.537943 | 7.4638688 | -4.034944 | 0.0002074 | 0.0033332 | down-regulated |
| MRC1 | -1.399147 | 8.2225391 | -4.026728 | 0.0002128 | 0.0033914 | down-regulated |
| SLC7A7 | -1.198116 | 7.7944496 | -4.026378 | 0.000213 | 0.0033914 | down-regulated |
| ALOX5 | -1.02225 | 10.06137 | -3.973561 | 0.0002511 | 0.0038127 | down-regulated |
| SEPP1 | -2.376083 | 9.0746604 | -3.956291 | 0.000265 | 0.0039635 | down-regulated |
| IRAK3 | -1.410925 | 5.9378171 | -3.951421 | 0.000269 | 0.0039943 | down-regulated |
| PLBD1 | -1.09644 | 7.9428737 | -3.947794 | 0.0002721 | 0.0040211 | down-regulated |
| HMOX1 | -1.044452 | 6.2688736 | -3.943326 | 0.0002759 | 0.0040567 | down-regulated |
| C1QB | -1.677793 | 6.5751283 | -3.903494 | 0.0003121 | 0.0044694 | down-regulated |
| RHOBTB1 | -1.203231 | 5.0490394 | -3.888932 | 0.0003264 | 0.0045903 | down-regulated |
| ZWINT | 1.0151844 | 7.1102249 | 3.8346037 | 0.0003857 | 0.0051846 | upregulated |
| CD163 | -1.345149 | 6.7887358 | -3.832907 | 0.0003878 | 0.0051937 | down-regulated |
| CD9 | -1.341699 | 8.0564495 | -3.794749 | 0.0004358 | 0.0056575 | down-regulated |
| TRIM36 | -1.218422 | 6.1653625 | -3.794735 | 0.0004358 | 0.0056575 | down-regulated |
| MAFB | -1.549326 | 9.21064 | -3.792265 | 0.0004391 | 0.005684 | down-regulated |
| MARCO | -1.224514 | 7.0892371 | -3.74171 | 0.0005121 | 0.006348 | down-regulated |
| PRKAR2B | -1.040648 | 6.3173139 | -3.739699 | 0.0005153 | 0.0063785 | down-regulated |
| PYGL | -1.126321 | 6.7822505 | -3.737343 | 0.000519 | 0.0064164 | down-regulated |
| FOS | -1.088599 | 7.8266477 | -3.642383 | 0.0006911 | 0.0079258 | down-regulated |
| ARHGAP22 | -1.035148 | 5.741065 | -3.640009 | 0.0006961 | 0.0079375 | down-regulated |
| PDGFC | -1.376155 | 7.6878724 | -3.636233 | 0.000704 | 0.0080189 | down-regulated |
| EGR1 | -1.188187 | 8.809015 | -3.573338 | 0.0008495 | 0.0090451 | down-regulated |
| CACNA2D3 | -1.038957 | 7.0849275 | -3.567248 | 0.000865 | 0.0091623 | down-regulated |
| LGMN | -1.088006 | 6.7839735 | -3.562611 | 0.000877 | 0.0092603 | down-regulated |
| GSN | -1.021725 | 7.982577 | -3.532573 | 0.0009587 | 0.0098061 | down-regulated |
| S100A8 | -1.544806 | 8.126796 | -3.487555 | 0.0010949 | 0.0107181 | down-regulated |
| CXCL2 | -1.233328 | 4.2656583 | -3.487549 | 0.001095 | 0.0107181 | down-regulated |
| SHCBP1 | 1.0247908 | 5.3631363 | 3.4552542 | 0.0012039 | 0.0114248 | upregulated |
| SLC2A5 | -1.398675 | 7.0440481 | -3.454037 | 0.0012082 | 0.0114442 | down-regulated |
| MS4A4A | -1.511608 | 6.1217867 | -3.395591 | 0.0014328 | 0.0129772 | down-regulated |
| EGR3 | -1.020386 | 5.6230695 | -3.376331 | 0.0015152 | 0.0133921 | down-regulated |
| AREG | -1.122283 | 8.5018553 | -3.371567 | 0.0015362 | 0.0135336 | down-regulated |
| ST18 | -1.015807 | 4.718939 | -3.210189 | 0.0024383 | 0.0186754 | down-regulated |
| C1QA | -1.395714 | 6.9677951 | -3.165346 | 0.0027667 | 0.0204794 | down-regulated |
| GPNMB | -1.458969 | 6.6516769 | -2.95487 | 0.0049461 | 0.0310329 | down-regulated |
|  |  |  |  |  |  |  |

Upregulated: upregulated in MS cases; down-regulated: down-regulated in MS cases.

| \| **Supplementary Table 3.** GSVA results of the KEGG gene-set enriched in samples of derivation dataset (MS Vs Control). \| \| \| \| \| \| \| \| \| --- \| --- \| --- \| --- \| --- \| --- \| --- \| --- \| \| Pathways \| logFC \| \| AveExpr \| t \| P.Value \| adj.P.Val \| \| KEGG_DNA_REPLICATION \| 0.4633983 \| \| 0.0129065 \| 6.259793 \| 8.75E-08 \| 1.54E-05 \| \| KEGG_MISMATCH_REPAIR \| 0.4097659 \| \| 0.0094595 \| 5.7419009 \| 5.56E-07 \| 9.73E-05 \| \| KEGG_PROTEIN_EXPORT \| 0.3732066 \| \| 0.0091541 \| 4.7139473 \| 1.99E-05 \| 0.00328783 \| \| KEGG_PROTEASOME \| \| 0.3639295 \| -0.001594 \| 4.4266529 \| 5.23E-05 \| 0.008415826 \| \| KEGG_CELL_CYCLE \| 0.362583 \| \| 0.0205624 \| 7.3254808 \| 1.90E-09 \| 3.40E-07 \| \| KEGG_ HOMOLOGOUS_RECOMBINATION \| 0.2932934 \| \| 0.0249985 \| 5.3443093 \| 2.26E-06 \| 0.000386659 \| \| KEGG_NUCLEOTIDE_EXCISION_REPAIR \| 0.2875194 \| \| 0.0195746 \| 5.1788253 \| 4.03E-06 \| 0.000681118 \| \| KEGG_BASAL_TRANSCRIPTION_FACTORS \| 0.2792698 \| \| 0.0158009 \| 4.8662075 \| 1.19E-05 \| 0.001980705 \| \| KEGG_BASE_EXCISION_REPAIR \| 0.2699845 \| \| 0.0161584 \| 4.4902766 \| 4.23E-05 \| 0.006851675 \| \| KEGG_UBIQUITIN_MEDIATED_PROTEOLYSIS \| 0.2573532 \| \| 0.0155627 \| 5.6721114 \| 7.12E-07 \| 0.000123168 \| \| KEGG_PRIMARY_IMMUNODEFICIENCY \| 0.2566642 \| \| 0.0313727 \| 4.2699972 \| 8.76E-05 \| 0.014023566 \| \| KEGG_RNA_DEGRADATION \| 0.2457069 \| \| 0.0199789 \| 4.1434413 \| 0.0001324 \| 0.02078393 \| \| KEGG_OOCYTE_MEIOSIS \| 0.2146053 \| \| 0.0131894 \| 5.3751023 \| 2.03E-06 \| 0.000349111 \| \| KEGG_ARRHYTHMOGENIC_RIGHT_VENTRICULAR  _CARDIOMYOPATHY_ARVC \| -0.20114 \| \| -0.006009 \| -4.662755 \| 2.37E-05 \| 0.003886264 \| \| KEGG_LYSOSOME \| -0.269958 \| \| -0.003762 \| -4.565477 \| 3.29E-05 \| 0.005359971 \| \| KEGG_GALACTOSE_METABOLISM \| -0.276211 \| \| -0.017543 \| -4.120363 \| 0.0001426 \| 0.022252693 \| \| KEGG_ALDOSTERONE_REGULATED_SODIUM  _REABSORPTION \| -0.278907 \| \| -0.007088 \| -6.31297 \| 7.23E-08 \| 1.28E-05 \| \| KEGG_SYSTEMIC_LUPUS_ERYTHEMATOSUS \| -0.300034 \| \| 0.0050466 \| -4.249144 \| 9.38E-05 \| 0.014920673 \| \| KEGG_ARACHIDONIC_ACID_METABOLISM \| -0.305608 \| \| -0.005328 \| -5.674655 \| 7.06E-07 \| 0.000122768 \| \| KEGG_ASTHMA \| -0.309598 \| \| 0.0026231 \| -4.014484 \| 0.0002005 \| 0.03107589 \| \| KEGG_GLYCOSPHINGOLIPID_BIOSYNTHESIS  _GLOBO_SERIES \| -0.321774 \| \| -0.019052 \| -5.229076 \| 3.38E-06 \| 0.000575115 \| \| KEGG_GLYCOSAMINOGLYCAN_DEGRADATION \| -0.323153 \| \| -0.014136 \| -5.152354 \| 4.42E-06 \| 0.00074238 \| \| KEGG_COMPLEMENT_AND_COAGULATION_CASCADES \| -0.363346 \| \| -0.002587 \| -6.865249 \| 9.93E-09 \| 1.77E-06 \| \|  \|  \| \|  \|  \|  \|  \|  \| |  |  |  |
| --- | --- | --- | --- | --- | --- | --- | --- | --- | --- | --- | --- | --- | --- | --- | --- | --- | --- | --- | --- | --- | --- | --- | --- | --- | --- | --- | --- | --- | --- | --- | --- | --- | --- | --- | --- | --- | --- | --- | --- | --- | --- | --- | --- | --- | --- | --- | --- | --- | --- | --- | --- | --- | --- | --- | --- | --- | --- | --- | --- | --- | --- | --- | --- | --- | --- | --- | --- | --- | --- | --- | --- | --- | --- | --- | --- | --- | --- | --- | --- | --- | --- | --- | --- | --- | --- | --- | --- | --- | --- | --- | --- | --- | --- | --- | --- | --- | --- | --- | --- | --- | --- | --- | --- | --- | --- | --- | --- | --- | --- | --- | --- | --- | --- | --- | --- | --- | --- | --- | --- | --- | --- | --- | --- | --- | --- | --- | --- | --- | --- | --- | --- | --- | --- | --- | --- | --- | --- | --- | --- | --- | --- | --- | --- | --- | --- | --- | --- | --- | --- | --- | --- | --- | --- | --- | --- | --- | --- | --- | --- | --- | --- | --- | --- | --- | --- | --- | --- | --- | --- | --- | --- | --- | --- | --- | --- | --- | --- | --- | --- | --- | --- | --- | --- | --- | --- | --- | --- |

|  |  |
| --- | --- |

| **Supplementary Table 4.** GSEA results of the most of the significantly altered pathways were activated in the derivation dataset. | | | | | |
| --- | --- | --- | --- | --- | --- |
| Pathways | SIZE | ES | NES | NOM p-val | LEADING EDGE |
| KEGG_UBIQUITIN_MEDIATED_PROTEOLYSIS | 103 | 0.4906007 | 1.6011993 | 0.01844262 | tags=32%, list=17%, signal=38% |
| 111KEGG_PRIMARY_IMMUNODEFICIENCY | 31 | 0.5857058 | 1.5473702 | 0.03238867 | tags=29%, list=8%, signal=32% |
| KEGG_ALDOSTERONE_REGULATED  _SODIUM_REABSORPTION | 35 | -0.566428 | -1.714932 | 0.00205339 | tags=43%, list=19%, signal=53% |
| KEGG_GLYCOSAMINOGLYCAN_DEGRADATION | 16 | -0.601555 | -1.683366 | 0.00594059 | tags=69%, list=29%, signal=97% |
| KEGG_REGULATION_OF_AUTOPHAGY | 27 | -0.497889 | -1.628215 | 0.03354298 | tags=33%, list=17%, signal=40% |
| KEGG_MAPK_SIGNALING_PATHWAY | 218 | -0.42415 | -1.624682 | 0.00581395 | tags=31%, list=18%, signal=37% |
| KEGG_LYSOSOME | 100 | -0.516343 | -1.565903 | 0.03853565 | tags=57%, list=29%, signal=80% |
| KEGG_AMYOTROPHIC_LATERAL_SCLEROSIS_ALS | 48 | -0.45074 | -1.560915 | 0.02376238 | tags=31%, list=14%, signal=36% |
| KEGG_TOLL_LIKE_RECEPTOR_SIGNALING_PATHWAY | 89 | -0.527447 | -1.544268 | 0.02509653 | tags=26%, list=11%, signal=29% |
| KEGG_COMPLEMENT_AND_COAGULATION_CASCADES | 65 | -0.678132 | -1.541361 | 0.00591716 | tags=42%, list=12%, signal=47% |
| KEGG_DILATED_CARDIOMYOPATHY | 77 | -0.438393 | -1.519755 | 0.02419355 | tags=31%, list=15%, signal=36% |
| KEGG_NOD_LIKE_RECEPTOR_SIGNALING_PATHWAY | 48 | -0.54695 | -1.502371 | 0.03508772 | tags=21%, list=8%, signal=23% |
| KEGG_REGULATION_OF_ACTIN_CYTOSKELETON | 160 | -0.383931 | -1.491755 | 0.01183432 | tags=26%, list=18%, signal=31% |
| KEGG_CARDIAC_MUSCLE_CONTRACTION | 56 | -0.43946 | -1.482024 | 0.04444445 | tags=43%, list=28%, signal=59% |
| KEGG_ARRHYTHMOGENIC_RIGHT_VENTRICULAR  _CARDIOMYOPATHY_ARVC | 60 | -0.451383 | -1.479208 | 0.00816327 | tags=38%, list=20%, signal=47% |
| KEGG_SYSTEMIC_LUPUS_ERYTHEMATOSUS | 49 | -0.648192 | -1.468508 | 0.03929273 | tags=41%, list=12%, signal=46% |
| KEGG_CYTOKINE_CYTOKINE_RECEPTOR_INTERACTION | 214 | -0.44225 | -1.458918 | 0.00592885 | tags=28%, list=13%, signal=31% |
| KEGG_GAP_JUNCTION | 73 | -0.439719 | -1.446544 | 0.03427419 | tags=33%, list=19%, signal=40% |
| KEGG_CHEMOKINE_SIGNALING_PATHWAY | 151 | -0.386116 | -1.405823 | 0.03762376 | tags=24%, list=17%, signal=28% |
| KEGG_PATHWAYS_IN_CANCER | 275 | -0.34469 | -1.347229 | 0.04373758 | tags=28%, list=21%, signal=35% |

| **Supplementary Table 5.** The differentially expressed genes of dataset E-MTAB-2374. | | | | | | |
| --- | --- | --- | --- | --- | --- | --- |
| Gene ID | logFC | AveExpr | t | P.Value | adj.P.Val | up/down-regulated |
| IGHG3 | 6.500772 | 10.965096 | 10.57058 | 2.80E-12 | 2.83E-08 | upregulated |
| POU2AF1 | 2.515576 | 11.191446 | 9.3575991 | 6.27E-11 | 2.44E-07 | upregulated |
| IGLJ3 | 2.6587556 | 8.7714687 | 9.3044394 | 7.22E-11 | 2.44E-07 | upregulated |
| IGHM | 3.1550798 | 9.2714968 | 8.4640073 | 7.00E-10 | 1.77E-06 | upregulated |
| SDC1 | 3.7375811 | 7.2943478 | 8.2492526 | 1.27E-09 | 2.57E-06 | upregulated |
| CD79A | 3.1042321 | 8.3950174 | 7.3204275 | 1.77E-08 | 2.99E-05 | upregulated |
| TNFRSF17 | 4.9517463 | 8.0427963 | 7.1749315 | 2.70E-08 | 3.91E-05 | upregulated |
| PNOC | 2.3853161 | 6.5910139 | 6.0903162 | 6.60E-07 | 0.0008357 | upregulated |
| OSBPL10 | 1.5224986 | 6.0552093 | 5.7475114 | 1.84E-06 | 0.0020692 | upregulated |
| CAV1 | 2.7302121 | 6.2138358 | 5.4876127 | 4.01E-06 | 0.0040556 | upregulated |
| CD24 | 1.9900198 | 6.7873868 | 5.4268216 | 4.81E-06 | 0.0044233 | upregulated |
| BTN1A1 | 2.6348674 | 4.6196998 | 5.2828124 | 7.40E-06 | 0.0062404 | upregulated |
| KIAA0125 | 3.0640851 | 6.6954887 | 5.2024613 | 9.40E-06 | 0.00652 | upregulated |
| CDCP1 | -1.40839 | 7.2730623 | -5.193572 | 9.66E-06 | 0.00652 | down-regulated |
| CD19 | 2.167206 | 6.7742304 | 5.1642348 | 1.05E-05 | 0.0066729 | upregulated |
| NECTIN2 | -1.507643 | 7.1723468 | -5.02123 | 1.62E-05 | 0.0090918 | down-regulated |
| ST6GALNAC2 | -1.208495 | 8.4833631 | -4.914926 | 2.22E-05 | 0.0118244 | down-regulated |
| ATP9A | 1.2111452 | 8.8073744 | 4.8925976 | 2.37E-05 | 0.0120052 | upregulated |
| MS4A1 | 1.9634373 | 8.0137595 | 4.8249687 | 2.90E-05 | 0.0131801 | upregulated |
| RBMS2 | -1.344061 | 5.7498855 | -4.823535 | 2.91E-05 | 0.0131801 | down-regulated |
| TNFRSF8 | -1.306044 | 7.7865271 | -4.801191 | 3.11E-05 | 0.0131801 | down-regulated |
| CMKLR1 | -1.388136 | 6.7836972 | -4.781276 | 3.30E-05 | 0.0131801 | down-regulated |
| COBLL1 | 1.2080878 | 4.456962 | 4.7457279 | 3.67E-05 | 0.0137575 | upregulated |
| TMEM176A | -1.783461 | 8.5647595 | -4.700344 | 4.20E-05 | 0.0141743 | down-regulated |
| PXDC1 | -1.10572 | 9.8329821 | -4.700103 | 4.20E-05 | 0.0141743 | down-regulated |
| P2RX5 | 1.1691477 | 9.866142 | 4.683004 | 4.42E-05 | 0.0143731 | upregulated |
| ACPP | -1.949109 | 6.720092 | -4.673597 | 4.54E-05 | 0.0143731 | down-regulated |
| BUB1 | 1.2173915 | 5.3725092 | 4.660848 | 4.72E-05 | 0.0144732 | upregulated |
| RAB20 | -1.49318 | 9.1136403 | -4.608193 | 5.51E-05 | 0.0164129 | down-regulated |
| CD79B | 1.2604604 | 8.7850715 | 4.5391583 | 6.76E-05 | 0.0186313 | upregulated |
| LILRA1 | -1.102453 | 7.4038244 | -4.530112 | 6.94E-05 | 0.0186313 | down-regulated |
| CD68 | -1.19163 | 7.5008501 | -4.527478 | 6.99E-05 | 0.0186313 | down-regulated |
| BMP2K | -1.066808 | 8.1766214 | -4.493357 | 7.73E-05 | 0.019569 | down-regulated |
| ALOX5 | -1.087519 | 9.0933631 | -4.446785 | 8.86E-05 | 0.0211409 | down-regulated |
| CD33 | -1.033725 | 9.9646278 | -4.364454 | 0.0001128 | 0.0212309 | down-regulated |
| AVPI1 | -1.259209 | 9.2477853 | -4.357457 | 0.0001151 | 0.0212309 | down-regulated |
| CD86 | -1.058162 | 10.544252 | -4.334 | 0.0001233 | 0.0212309 | down-regulated |
| SERPING1 | -1.325517 | 8.4817405 | -4.313188 | 0.000131 | 0.0212309 | down-regulated |
| RRM2 | 2.6218224 | 8.4374059 | 4.3111034 | 0.0001318 | 0.0212309 | upregulated |
| CEBPD | -1.063342 | 10.353454 | -4.287607 | 0.0001412 | 0.0212309 | down-regulated |
| TCL1A | 2.001458 | 7.5269287 | 4.2850111 | 0.0001423 | 0.0212309 | upregulated |
| OSBPL1A | -1.351032 | 7.5647228 | -4.274457 | 0.0001467 | 0.0215284 | down-regulated |
| IL12B | 1.7788662 | 3.3240665 | 4.2650375 | 0.0001508 | 0.0218108 | upregulated |
| DOCK4 | -1.550047 | 9.7567336 | -4.242932 | 0.0001608 | 0.0222183 | down-regulated |
| CSF1R | -1.331567 | 11.411542 | -4.240982 | 0.0001617 | 0.0222183 | down-regulated |
| DLGAP5 | 2.2878601 | 5.9632235 | 4.2117592 | 0.000176 | 0.0222183 | upregulated |
| DFNA5 | -2.070516 | 8.3119975 | -4.208444 | 0.0001777 | 0.0222183 | down-regulated |
| NFIL3 | -1.000959 | 9.6281532 | -4.15575 | 0.000207 | 0.024029 | down-regulated |
| TLR2 | -1.245801 | 9.6911967 | -4.140113 | 0.0002166 | 0.024029 | down-regulated |
| PDGFC | -1.28948 | 9.5801997 | -4.138803 | 0.0002174 | 0.024029 | down-regulated |
| IL13RA1 | -1.022265 | 9.0364097 | -4.121483 | 0.0002286 | 0.0243665 | down-regulated |
| CDC20 | 1.6412491 | 7.0373544 | 4.1007242 | 0.0002427 | 0.0248286 | upregulated |
| CD38 | 1.8117091 | 7.4003508 | 4.094924 | 0.0002468 | 0.0248286 | upregulated |
| HYMAI | -1.566063 | 4.455693 | -4.075486 | 0.000261 | 0.0248286 | down-regulated |
| STIL | 1.1931714 | 8.1867459 | 4.0730027 | 0.0002629 | 0.0248286 | upregulated |
| SLC2A5 | -1.486302 | 10.003048 | -4.071086 | 0.0002644 | 0.0248286 | down-regulated |
| HGF | -1.027084 | 5.241726 | -4.068727 | 0.0002662 | 0.0248286 | down-regulated |
| GAREM1 | -1.14 | 6.1107033 | -4.063889 | 0.0002699 | 0.0248286 | down-regulated |
| SNX7 | -1.901117 | 7.3874491 | -4.060299 | 0.0002727 | 0.0248286 | down-regulated |
| PDLIM1 | 1.4136147 | 8.9315352 | 4.0490216 | 0.0002817 | 0.0248286 | upregulated |
| RPGRIP1 | -1.273607 | 6.5782446 | -4.028317 | 0.0002989 | 0.0254309 | down-regulated |
| BIRC5 | 1.190982 | 6.6758961 | 4.0105346 | 0.0003146 | 0.0257837 | upregulated |
| KIAA0101 | 1.0662617 | 6.5680332 | 4.0092573 | 0.0003157 | 0.0257837 | upregulated |
| LILRA2 | -1.039144 | 8.5120462 | -4.005373 | 0.0003193 | 0.0258638 | down-regulated |
| PRKAR2B | -1.261486 | 7.6142155 | -3.999327 | 0.0003249 | 0.0261071 | down-regulated |
| SLC7A11 | 1.1904189 | 5.110917 | 3.9904051 | 0.0003333 | 0.0262503 | upregulated |
| MMP2 | -1.606705 | 7.9339498 | -3.981883 | 0.0003415 | 0.0263969 | down-regulated |
| BLM | 1.1575625 | 8.6446147 | 3.9637989 | 0.0003596 | 0.0269746 | upregulated |
| HBEGF | -1.642028 | 8.6325732 | -3.935386 | 0.00039 | 0.0281016 | down-regulated |
| C2 | -1.163906 | 7.5365442 | -3.927453 | 0.0003989 | 0.028443 | down-regulated |
| AREG | -1.741789 | 6.8625644 | -3.889778 | 0.0004441 | 0.02853 | down-regulated |
| LMO2 | -1.019522 | 10.472375 | -3.889539 | 0.0004444 | 0.02853 | down-regulated |
| NRGN | -1.268671 | 6.432326 | -3.88924 | 0.0004448 | 0.02853 | down-regulated |
| FCGBP | -1.135206 | 12.136379 | -3.885928 | 0.000449 | 0.02853 | down-regulated |
| TMEM45A | 2.1997524 | 6.3532177 | 3.8696572 | 0.0004703 | 0.0292885 | upregulated |
| CR2 | 1.1121662 | 4.1548804 | 3.8621727 | 0.0004804 | 0.0292885 | upregulated |
| SIRPA | -1.019577 | 9.2919352 | -3.849027 | 0.0004986 | 0.0292885 | down-regulated |
| CAMSAP2 | -1.218684 | 7.5155683 | -3.840196 | 0.0005112 | 0.0292885 | down-regulated |
| MOK | -1.0938 | 5.9366997 | -3.836515 | 0.0005166 | 0.0292885 | down-regulated |
| IL1B | -1.447641 | 8.6799461 | -3.834274 | 0.0005199 | 0.0292885 | down-regulated |
| DRAM1 | -1.09637 | 10.235145 | -3.830635 | 0.0005253 | 0.0292885 | down-regulated |
| HJURP | 1.2369972 | 5.4380954 | 3.8207833 | 0.0005401 | 0.0294047 | upregulated |
| ENG | -1.064048 | 9.4045325 | -3.8122 | 0.0005534 | 0.0296358 | down-regulated |
| MYO7A | -1.077576 | 6.6152389 | -3.808348 | 0.0005595 | 0.0296358 | down-regulated |
| PILRA | -1.162947 | 10.580484 | -3.808002 | 0.00056 | 0.0296358 | down-regulated |
| LILRB2 | -1.147991 | 10.046081 | -3.781941 | 0.0006028 | 0.0308922 | down-regulated |
| PLAUR | -1.145932 | 9.1399962 | -3.781175 | 0.0006041 | 0.0308922 | down-regulated |
| FPR1 | -1.303165 | 7.4560285 | -3.770936 | 0.0006217 | 0.0314788 | down-regulated |
| SLC11A1 | -1.355296 | 9.6175355 | -3.769019 | 0.0006251 | 0.0314918 | down-regulated |
| EPB41L3 | -1.514481 | 8.3002169 | -3.764012 | 0.000634 | 0.0316244 | down-regulated |
| SDC3 | -1.482502 | 8.1335199 | -3.760202 | 0.0006408 | 0.0317003 | down-regulated |
| HIST1H1D | 1.0570821 | 9.6566452 | 3.756422 | 0.0006477 | 0.0318369 | upregulated |
| LTBR | -1.056268 | 7.5373874 | -3.750085 | 0.0006593 | 0.0320804 | down-regulated |
| ADAP2 | -1.269936 | 9.6593037 | -3.746877 | 0.0006653 | 0.0320804 | down-regulated |
| DOCK1 | -1.139688 | 5.8653966 | -3.742442 | 0.0006737 | 0.032329 | down-regulated |
| RTN1 | -1.002148 | 9.8445456 | -3.718999 | 0.0007195 | 0.0330639 | down-regulated |
| GLDC | 1.310398 | 4.0369558 | 3.7186413 | 0.0007202 | 0.0330639 | upregulated |
| NLRP3 | -1.015208 | 7.7673223 | -3.717949 | 0.0007216 | 0.0330639 | down-regulated |
| HPGDS | -1.462531 | 8.8346028 | -3.711286 | 0.0007352 | 0.0335357 | down-regulated |
| CYP27A1 | -1.402362 | 10.439297 | -3.695306 | 0.0007689 | 0.0346037 | down-regulated |
| SCN3A | 2.0591048 | 3.5064708 | 3.6662902 | 0.0008339 | 0.0362168 | upregulated |
| ARHGAP6 | -1.070365 | 5.5159174 | -3.663456 | 0.0008405 | 0.0362168 | down-regulated |
| TMEM51 | -1.155433 | 8.6761968 | -3.66086 | 0.0008466 | 0.0362379 | down-regulated |
| FBLN5 | 1.2144887 | 7.9834017 | 3.655122 | 0.0008603 | 0.0362969 | upregulated |
| HMOX1 | -1.263574 | 9.0875904 | -3.647805 | 0.000878 | 0.0365422 | down-regulated |
| HOXB7 | -1.511725 | 6.7079168 | -3.646779 | 0.0008805 | 0.0365422 | down-regulated |
| TRIM36 | -1.087255 | 7.7586865 | -3.641118 | 0.0008945 | 0.0367202 | down-regulated |
| CEBPA | -1.185808 | 9.8153658 | -3.639105 | 0.0008996 | 0.0367301 | down-regulated |
| CSF3R | -1.307996 | 7.7419846 | -3.636041 | 0.0009073 | 0.0368961 | down-regulated |
| KCNN3 | 1.3680072 | 5.1255027 | 3.609665 | 0.0009764 | 0.0378148 | upregulated |
| DTL | 1.5989314 | 5.9184492 | 3.6084194 | 0.0009797 | 0.0378148 | upregulated |
| APOBEC3B | 1.759902 | 5.7749742 | 3.6070239 | 0.0009835 | 0.0378148 | upregulated |
| OLR1 | -1.410561 | 10.064037 | -3.607001 | 0.0009836 | 0.0378148 | down-regulated |
| HLA-DOB | 1.0531259 | 8.4830298 | 3.6034488 | 0.0009934 | 0.0378148 | upregulated |
| F13A1 | -1.283373 | 10.837257 | -3.590862 | 0.0010287 | 0.0384365 | down-regulated |
| JAM2 | -1.550763 | 7.46988 | -3.563682 | 0.0011091 | 0.0399777 | down-regulated |
| PALLD | -1.056755 | 8.4566243 | -3.554903 | 0.0011364 | 0.040159 | down-regulated |
| TSPAN13 | 1.0372986 | 10.028321 | 3.5546607 | 0.0011371 | 0.040159 | upregulated |
| TPX2 | 1.3304763 | 6.4608083 | 3.5487914 | 0.0011557 | 0.040371 | upregulated |
| CD22 | 1.1024691 | 5.9621862 | 3.5486415 | 0.0011562 | 0.040371 | upregulated |
| PKIG | 1.0457903 | 7.211642 | 3.5419798 | 0.0011777 | 0.0405377 | upregulated |
| POMZP3 | -1.020275 | 8.5983307 | -3.53728 | 0.001193 | 0.0407336 | down-regulated |
| BUB1B | 1.333695 | 7.0504001 | 3.5361334 | 0.0011968 | 0.0407336 | upregulated |
| ARHGAP22 | -1.108348 | 10.296732 | -3.535545 | 0.0011988 | 0.0407336 | down-regulated |
| C1QB | -1.146578 | 11.171772 | -3.513766 | 0.0012729 | 0.0415789 | down-regulated |
| MYB | 1.4086631 | 9.2089515 | 3.507737 | 0.0012942 | 0.0420242 | upregulated |
| CD300C | -1.072217 | 7.5881811 | -3.495543 | 0.0013383 | 0.0425513 | down-regulated |
| NEIL3 | 1.5728389 | 4.3454455 | 3.4918618 | 0.0013519 | 0.0425612 | upregulated |
| TPD52 | 1.529319 | 7.1305319 | 3.4847905 | 0.0013784 | 0.0429894 | upregulated |
| SLC15A3 | -1.0224 | 9.3362769 | -3.474815 | 0.0014167 | 0.0434367 | down-regulated |
| SNHG3 | 1.7315984 | 6.5224028 | 3.4718871 | 0.0014281 | 0.0434367 | upregulated |
| STEAP3 | -1.501713 | 9.0570161 | -3.4718 | 0.0014284 | 0.0434367 | down-regulated |
| DSC2 | -1.205021 | 5.4100886 | -3.463297 | 0.0014621 | 0.0440324 | down-regulated |
| STK32B | -1.309767 | 6.9444683 | -3.46247 | 0.0014654 | 0.0440324 | down-regulated |
| C1QA | -1.285648 | 11.106897 | -3.447825 | 0.0015253 | 0.0448478 | down-regulated |
| VSIG4 | -1.021105 | 11.49907 | -3.442987 | 0.0015457 | 0.0450604 | down-regulated |
| PTAFR | -1.020558 | 7.6560776 | -3.44161 | 0.0015515 | 0.0450604 | down-regulated |
| RGL1 | -1.070494 | 9.8448152 | -3.441106 | 0.0015536 | 0.0450604 | down-regulated |
| TLR5 | -1.185304 | 8.5293156 | -3.428163 | 0.0016095 | 0.0461696 | down-regulated |
| CXCL12 | -1.308623 | 6.4878867 | -3.41808 | 0.0016544 | 0.0467335 | down-regulated |
| SAMD4A | -1.013302 | 7.1363268 | -3.413728 | 0.0016741 | 0.0470892 | down-regulated |
| FSCN1 | -1.11665 | 10.429365 | -3.409014 | 0.0016958 | 0.0474342 | down-regulated |
| RNASE3 | -1.638788 | 7.1858592 | -3.406709 | 0.0017064 | 0.0476012 | down-regulated |
| VCAN | -1.153053 | 8.6035713 | -3.404978 | 0.0017145 | 0.0476947 | down-regulated |
| MARCO | -1.422871 | 10.047434 | -3.394375 | 0.0017647 | 0.0488221 | down-regulated |
| TRH | -1.693299 | 7.9742213 | -3.391232 | 0.0017798 | 0.0491068 | down-regulated |
| ENPP1 | -1.224404 | 5.9730105 | -3.388529 | 0.0017929 | 0.0493344 | down-regulated |
| EPS8 | -1.266065 | 9.8196388 | -3.387244 | 0.0017992 | 0.0493728 | down-regulated |
| FUT8 | 1.1650069 | 7.8957173 | 3.38483 | 0.001811 | 0.0494759 | upregulated |
| RNASE2 | -1.30479 | 11.153923 | -3.384486 | 0.0018127 | 0.0494759 | down-regulated |
|  |  |  |  |  |  |  |

Upregulated: upregulated in MS cases; down-regulated: down-regulated in MS cases.
